# Integration analysis of ATAC-seq and RNA-seq provides insight into fatty acid biosynthesis in *Schizochytrium limacinum* under nitrogen limitation stress

**DOI:** 10.1186/s12864-024-10043-5

**Published:** 2024-02-05

**Authors:** Duo Chen, Jing Chen, Rongchun Dai, Xuehai Zheng, Yuying Han, Youqiang Chen, Ting Xue

**Affiliations:** The Public Service Platform for Industrialization Development Technology of Marine Biological Medicine and Products of the State Oceanic Administration, Center of Engineering Technology Research for Microalga Germplasm Improvement of Fujian, Fujian Key Laboratory of Special Marine Bioresource Sustainable Utilization, College of Life Sciences, Fujian Normal University, Fuzhou, China

**Keywords:** *Schizochytrium limacinum*, Fatty acids, DHA, Chromatin accessibility, Gene expressions

## Abstract

**Background:**

*Schizochytrium limacinum* holds significant value utilized in the industrial-scale synthesis of natural DHA. Nitrogen-limited treatment can effectively increase the content of fatty acids and DHA, but there is currently no research on chromatin accessibility during the process of transcript regulation. The objective of this research was to delve into the workings of fatty acid production in *S. limacinum* by examining the accessibility of promoters and profiling gene expressions.

**Results:**

Results showed that differentially accessible chromatin regions (DARs)-associated genes were enriched in fatty acid metabolism, signal transduction mechanisms, and energy production. By identifying and annotating DARs-associated motifs, the study obtained 54 target transcription factor classes, including BPC, RAMOSA1, SPI1, MYC, and MYB families. Transcriptomics results revealed that several differentially expressed genes (DEGs), including *SlFAD2*, *SlALDH*, *SlCAS1*, *SlNSDHL*, and *SlDGKI*, are directly related to the biosynthesis of fatty acids, meanwhile, *SlRPS6KA, SlCAMK1, SlMYB3R1,* and *SlMYB3R5* serve as transcription factors that could potentially influence the regulation of fatty acid production. In the integration analysis of DARs and ATAC-seq, 13 genes were identified, which were shared by both DEGs and DARs-associated genes, including *SlCAKM*, *SlRP2*, *SlSHOC2*, *SlTN*, *SlSGK2*, *SlHMP*, *SlOGT*, *SlclpB*, and *SlDNAAF3*.

**Conclusions:**

*SlCAKM* may act as a negative regulator of fatty acid and DHA synthesis, while *SlSGK2* may act as a positive regulator, which requires further study in the future. These insights enhance our comprehension of the processes underlying fatty acid and DHA production in *S. limacinum*. They also supply a foundational theoretical framework and practical assistance for the development of strains rich in fatty acids and DHA.

**Supplementary Information:**

The online version contains supplementary material available at 10.1186/s12864-024-10043-5.

## Background

Thraustochytrids, including Schizochytrium and Thraustochytrium [1], are found in diverse marine environments, ranging from tropical to Antarctic waters. These organisms thrive from the ocean's surface to depths of up to 2000 m in nutrient-rich areas enriched by decaying organic matter. Their habitats span from seabed sediments to mangrove ecosystems and sewage outfalls. This abundance of nutrients fosters their heterotrophic lifestyle, making them integral to the decomposition of organic materials and the carbon cycle [2]. Within the mangrove forests, the leaf litter provides a conducive environment for growth and acts as a nutrient source, particularly for *Schizochytrium limacinum*, a member of the Cystachyclaceae family that relies on organic materials for survival [3]. In these ecosystems, chytrid fungi, algae, and various bacterial organisms serve as decomposers. Notably, chytrid fungi and algae differentiate themselves from bacteria by their ability to secrete a range of extracellular enzymes, such as proteases, esterases, cellulases, and chitinases, enhancing their decomposition capabilities [4]. Docosahexaenoic acid (DHA), an essential unsaturated fatty acid, is crucial for lowering cholesterol and triglyceride levels and preventing cardiovascular diseases like atherosclerosis, cerebral thrombosis, cerebral hemorrhage, and hypertension. It holds significant nutritional and medicinal importance [5–7]. Traditionally sourced from fish oil, the DHA concentration is subject to variability due to factors like fish species, location, season, and origin, leading to instability in its content. The fish oil production process is complex, and its products often have a fishy odor and are prone to contamination by marine pollutants [8, 9]. Microalgae and fungus production of DHA presents a practical solution to the limitations faced with fish oil resources. Microalgae and fungus cultivation boasts advantages such as short growth cycles, high yields, and a straightforward process for fatty acid extraction and purification. Unaffected by seasonal and regional constraints, this method transcends the traditional boundaries of fish oil applications. Therefore, the use of marine microalgae and fungus for DHA production offers expansive application prospects. Algal and fungus oil products enriched with DHA can significantly enhance brain function and improve cardiovascular and cerebrovascular health, yielding substantial economic and social benefits. In this context, *S. limacinum* stands out as an efficient natural synthesizer of DHA and an ideal species for its large-scale and commercial application. The use of acetate as a carbon source in cultures like *S. limacinum* B4D1 can further increase the DHA content in fatty acids.

Transcriptomics revealed that acetate promoted genes enhancing acetyl-CoA and the PKS pathway, boosting DHA. Altered gene activities increased saturated fatty acid β-oxidation and decreased polyunsaturated β-oxidation, leading to a higher DHA content [10]. Using waste-derived volatile fatty acids, *S. limacinum* could yielded biomass with 46.3% DHA, indicating potential industrial applications for DHA production [11]. These studies suggest that the synthesis of fatty acids and DHA can be regulated through substrates. Our previous research found that under nitrogen stress, *S. limacinum* could store more lipids, leading to increased fatty acid and DHA content, which trait was valuable for boosting commercial DHA yields under controlled nitrogen stress. We previously assembled the genome of *S. limacinum* SR21, with a size of 63 Mb and scaffold N50 of 2.67 Mb, which is nearly 20 times higher than the quality of other reported assembled genomes. We pinpointed two unigenes that encode desaturase (1–4 desaturase, 1–5 desaturase) and four unigenes encoding elongase protein (1–1 elongase, 1–3 elongase, 1–4 elongase, 1–6 elongase). In addition, all requisite genes for the FAS pathway in map00062 and map01212 were annotated. Among the six regulatory genes for very-long-chain fatty acid synthesis, we detected 1–6 elongase and 1–4 desaturase, along with several transcription factors, to have a high positive correlation with fatty acids and DHA accumulation. These included three members of the protein kinase family (schi20066080, schi20050050, and schi20028430), 2 MYB variants (schi20061500 and schi20062190), and 1 Zinc Finger (schi20050340) [12]. MYB genes were found to regulate plant development and synthesis of secondary metabolites [13, 14]. We also found that docosahexaenoic acid production by *Schizochytrium sp*. could be regulated by nitrogen limitation in culture media [15], and nitrogen limitation had a significant effect on the metabolism of DHA in *S. limacinum*. We identified several genes and TFs associated with the biosynthesis pathways of DHA by integrating analyses for genome and transcriptome data. While these investigations have initially examined the transcriptional control mechanisms of DHA in *S. limacinum* preliminarily, it is still unknown whether chromatin remodeling is related to DHA biosynthesis and how it responds to nitrogen limitation-mediated culture.

ATAC-seq, a technique for examining chromatin accessibility, stands out for its ability to identify regions where chromatin is readily bound and opened by the Tn5 transposase enzyme, followed by sequencing the DNA captured by Tn5. This method has gained prominence over alternatives like ChIP-seq, DNase-seq, and FAIRE-seq due to its minimal cell requirement, simplicity, and effectiveness in assessing whole-genome chromatin openness. In this study, we utilized ATAC-seq to pinpoint key differentially accessible chromatin regions (DARs), motifs, and transcription factors (TFs) that influence DHA accumulation in *S. limacinum* under nitrogen-limited culture conditions. Complementing this, RNA-seq provided a gene expression profile during this period, offering a valuable reference for deciphering the regulatory mechanisms of DHA synthesis in *S. limacinum*. By integrating ATAC-seq with RNA-seq data, we could delve into the regulatory and expression patterns of differentially expressed genes (DEGs) and their associated chromatin open regions. This approach enabled us to identify crucial regulatory elements driving DHA production in *S. limacinum*. This exploration into *S. limacinum*’s chromatin accessibility and its linkage to gene regulation is a novel approach, especially in the context of fatty acid and DHA production. Given the microalga and fungus commercial significance in natural DHA production, enhancing its yield is globally important. Our research into chromatin accessibility and gene expression has highlighted specific genes and transcription factors that are likely key players in fatty acid biosynthesis. This expands our understanding of the genetic and epigenetic mechanisms underlying DHA production. By correlating DARs with essential genes involved in lipid metabolism, energy conversion, and signal transduction, we have shed light on a sophisticated molecular network geared towards optimizing fatty acid synthesis. While providing immediate insights, this study also lays the groundwork for future research, particularly considering the potential regulatory roles of the identified genes in DHA production.

## Results

### Fatty acids and DHA accumulation of *S. limacinum* with different nitrogen supply

**Fig. 1:**
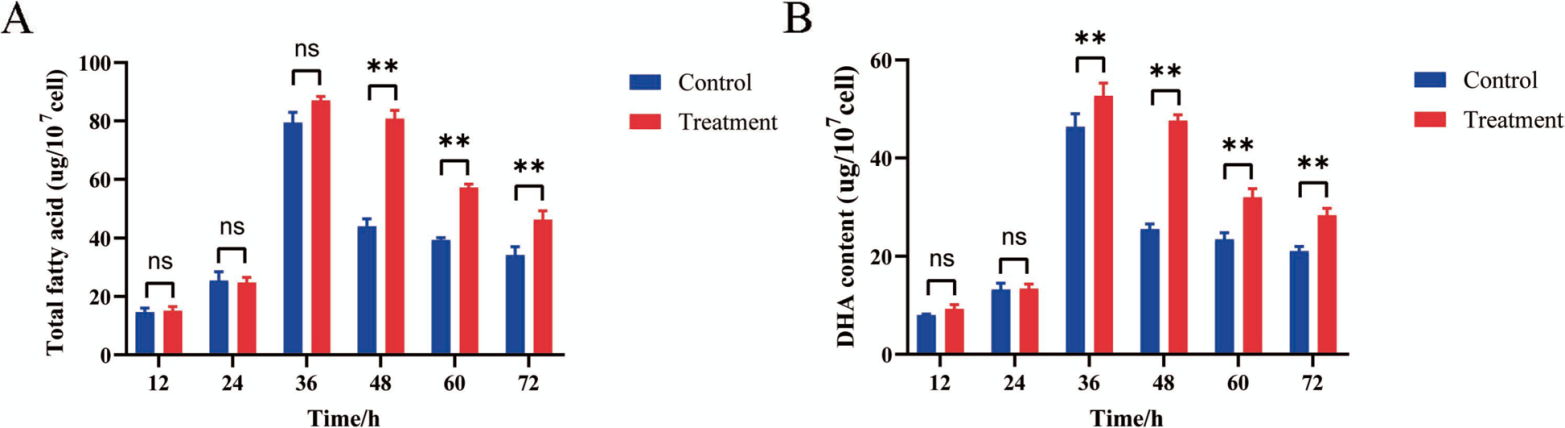
The variations in fatty acids and DHA content across different fermentation stages. **A** The total fatty acid content during culture. **B** DHA content during culture. Note: the error bars in the figure depict the standard deviation derived from three biological replicates. The asterisk (*) denotes a significant difference at *P* < *0.05*, while the double asterisk (**) indicates a significant difference at *P* < *0.01*

We observed that the fatty acid and DHA content increased gradually before 36 h of culture and then decreased gradually. The maximum difference between nitrogen-limited treatment and control was found at 48 h. The total fatty acid and DHA yield of *S. limacinum* in per unit cell, in nitrogen-limited for 48 h (total fatty acid content: 80.75 ug/10^7^cell; DHA content: 47.63 ug/10^7^cell) increased by 1.67-fold change (for total fatty acid content) and 1.86-fold change (for DHA content) compared to control treatments (total fatty acid content: 44.00 ug/10^7^cell; DHA content: 25.56 ug/10^7^cell), respectively (Fig. 1 A, B). These findings suggest that under nitrogen-deprived circumstances, there can be a substantial augmentation in the total fatty acid and DHA content of *S. limacinum*. At 48 h, the difference of total fatty acid and DHA content between the nitrogen-limited treatment and the control group was the most significant, which represents the pivotal phase for the buildup of fatty acid and DHA in *S. limacinum*. To effectively identify significant differences in chromosome accessibility, ATAC-seq study was conducted by sampling at 48-h time points.

### Identification and annotation of chromatin accessibility

**Fig. 2:**
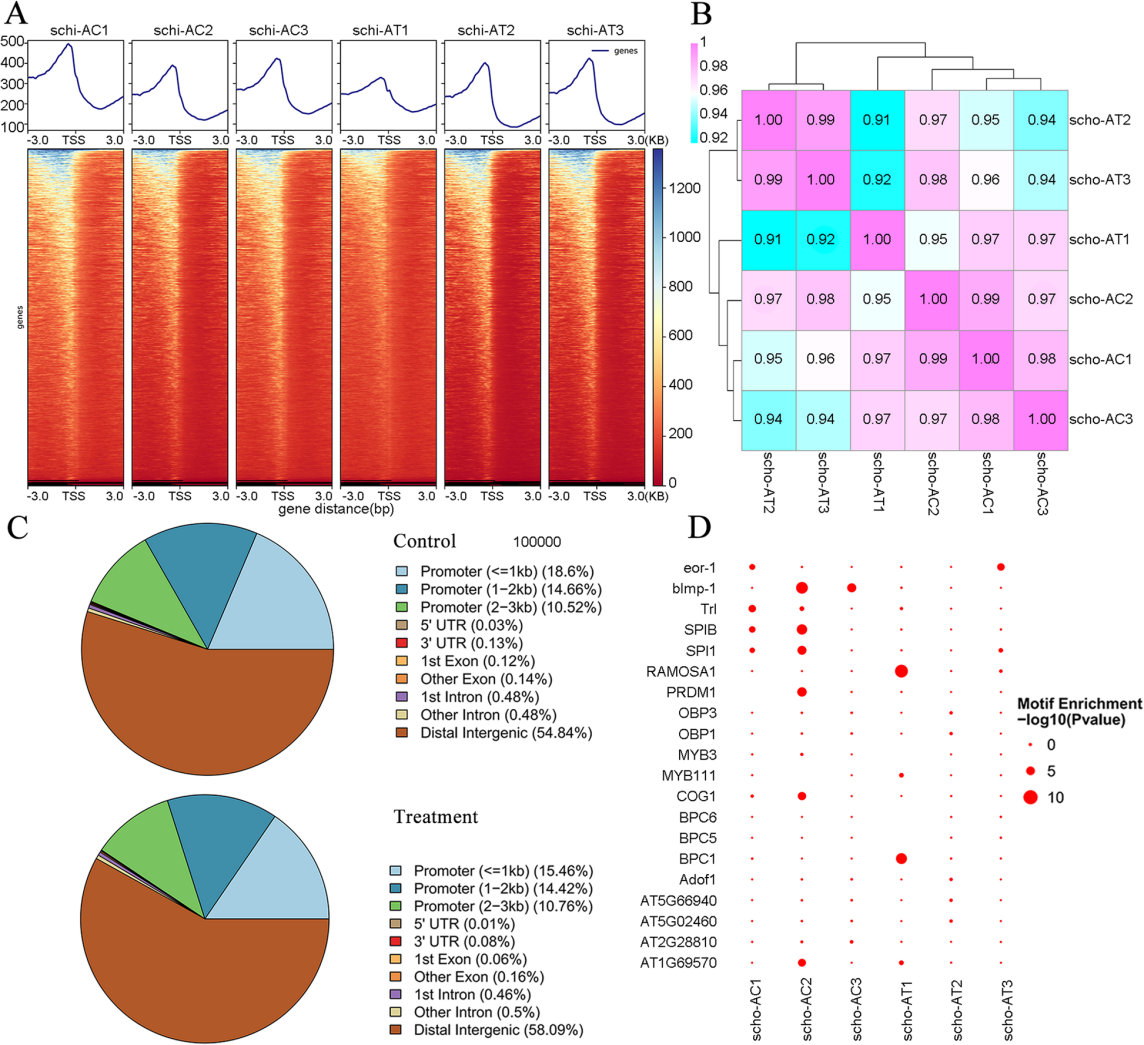
Changes and distribution of chromatin accessibility regions. **A** Distribution density map of ATAC data near the TSS. **B** The heat map displays the clustering of abundance values for sample reads using the Pearson correlation coefficient. **C** The distribution of open chromatin regions' accessibility is examined across different genomic regions in both the control and treatment conditions. **D** Enrichment diagram of transcription factor motifs

Six libraries were generated from three biological replicates each under control and nitrogen-limited treatment conditions to analyze the genomic chromatin accessibility landscape using ATAC-seq. The sequencing process produced 635.23 million high-quality reads with Q30 ≥ 92.73%, 99.91% of which successfully aligned with the *S. limacinum* genome (Table S1). In addition, over 93.36% of these clean reads were uniquely associated with the reference genome, but instances of duplication were also observed, which is the percentage of redundant reads among all clean reads, was less than 42.05%. Notably, we observed no reads that mapped to the mitochondrial genome, suggesting that the quality of the ATAC-seq data was reliable (Table S2). A density distribution heatmap demonstrated that the data was enriched in the proximity of TSS, thereby affirming the suitability of the data for subsequent analyses (Fig. 2A). Pearson correlation analysis showed six samples were all close to 1 (0.91–0.99), indicating that the similarities of expression patterns between the samples were quite high (Fig. 2B). We performed peak calling and found that the distal intergenic region had the largest proportion of peaks (54.87% to 58.63%), meanwhile, the share of peaks situated in the promoter region (≤ 3 kb) proximate to the transcription start site (TSS) varied between 40.11% and 43.78% (Fig. 2C; Table S3). Enrichment analysis through GO and KEGG indicated that a considerable portion of the genes associated with these peaks participated in processes such as metabolic activity (GO:0008152), biological regulation (GO:0008150), cellular functions (GO:0009987), response to stimuli (GO:0050896), carbon metabolism (ko01200), amino acid biosynthesis (ko01230), protein handling in the endoplasmic reticulum (ko04141), fatty acid breakdown (ko00071), and fatty acid metabolism (ko01212) (Figures S1-S2). Enrichment results of motifs from all samples and known TFs in the database showed that the top 20 enriched motifs of TFs mainly belong to the BPC (*BPC1*, *BPC5*, and *BPC6*), MYB (*MYB3*, *MYB111*), and OBP (*OBP1* and *OBP3*) families (Fig. 2D).

### Differential chromatin accessibility under nitrogen-limited culture

**Fig. 3:**
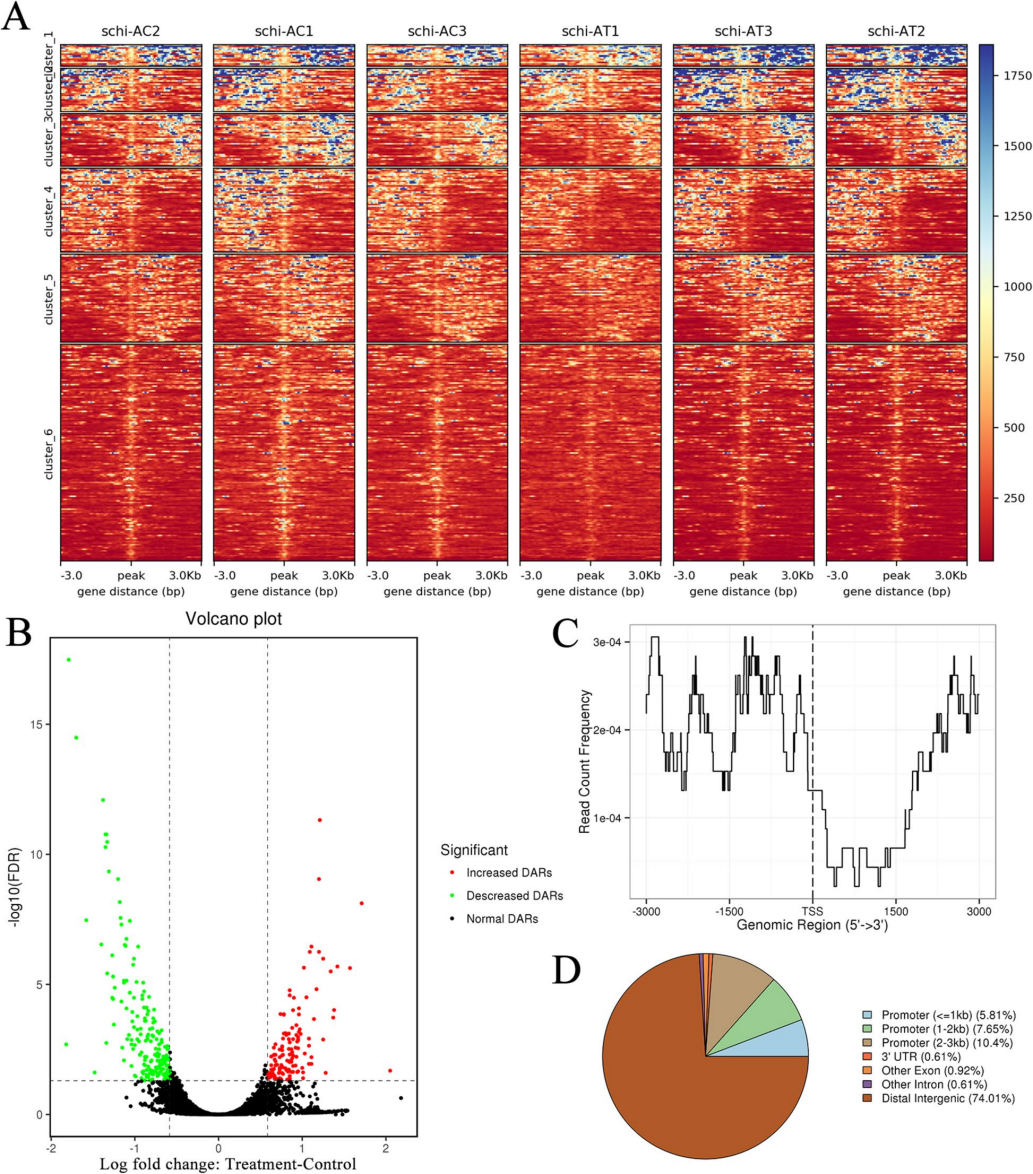
Identification of DARs-associated gene and motif. **A** The heat map displays the clustering of differential peaks observed in both the control and treatment conditions. **B** The volcano plot represents the differential peaks observed in the control vs. treatment comparison. **C** The average profile illustrates the binding of ChIP peaks to the transcription start site (TSS) region in both the control and treatment conditions. **D** The annotation classification map categorizes the differential peaks observed in the control vs. treatment analysis

Cluster heatmap of peak differences in each sample showed that groups the differential peaks for easy observation of the differences (Fig. 3A). A volcano plot was also created to represent the depot-specific open chromatin regions between control and treatment groups (Fig. 3B). Comparing the control versus treatment conditions, we identified 19,991 differential peaks in total, and from these, 327 differential accessibility regions (DARs) were discerned. Among these DARs, 132 exhibited increased regulation while 195 displayed downregulation. Of these, 23.5% were promoter (≤ 3kb) and 74.01% were distal intergenic (Fig. 3C and D). GO analysis indicated that the genes associated with the differential accessibility regions (DARs) primarily participated in various biological processes, including oxidation–reduction processes (GO:0055114), sphingolipid metabolic processes (GO:0006665), and regulation of apoptotic processes (GO:0042981), regulation of programmed cell death (GO:0042981), and membrane lipid metabolic process (GO:0006643) (Figure S3A). Regarding the cellular component, the majority of genes were found to be localized in the integral component of the membrane (GO:0016021) and cytosol (GO:0005829). In terms of molecular function, these genes were involved in activities such as oxidoreductase activity, specifically acting on CH-OH group donors (GO:0016614), flavin adenine dinucleotide binding (GO:0050660), and manganese ion binding (GO:0030145) (Figure S3B). Through KEGG enrichment analysis, it was observed that a number of genes were engaged in glycine, serine, and threonine metabolism (ko00260), ABC transporters (ko02010), and peroxisome (ko04146). Additionally, other genes were linked to fatty acid metabolism (ko01212), fatty acid degradation (ko00071), and biosynthesis of unsaturated fatty acids (ko01040), which are pivotal in the synthesis of fatty acids and DHA (Figure S4). After identifying and annotating motifs associated with DARs using MEME-ChIP software [16], we obtained 989 TF peaks that clustered into 54 TF classes. Among these, several TFs were from Trl, BPC6, RAMOSA1, SPI1, MYC, and MYB families, and some were closely associated with functional genes that play crucial roles in various aspects of fatty acid biology, including metabolism, degradation, and the biosynthesis of unsaturated fatty acids (Table S4).

### Transcriptome profile changing under nitrogen-limited treatment

**Fig. 4:**
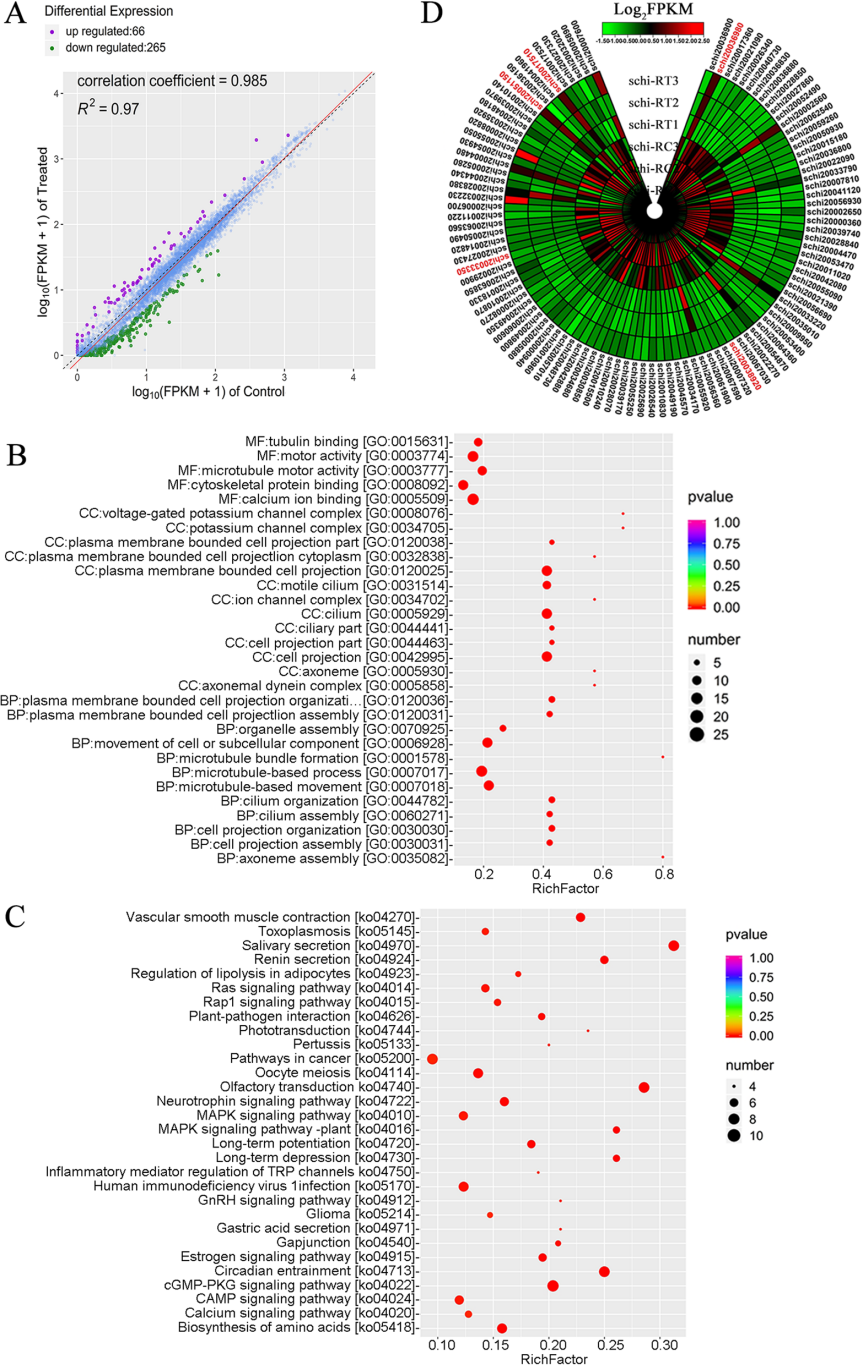
Depicts the analysis of differential expression and functional enrichment based on the RNA-seq data. **A** Differential expression dotplot in control vs. treatment. **B** GO enrichment pathways for DEGs in control vs. treatment. **C** KEGG enrichment pathways for DEGs in control vs. treatment. **D** Heatmap of DEGs visualizing the changes in the expression profiles in control vs. treatment

Pairwise comparison analysis of 48 h in nitrogen-limited revealed a total of 331 differentially expressed genes (DEGs), with 66 up-regulated and 265 down-regulated (Fig. 4A). We also identified 5 differentially expressed transcription factors (TFs) between control and treatment, including *SlbZIP* (schi20021090), *SlC3H* (schi20044680), *SlMYBs* (schi20033350, schi20041240), *SlWRKY* (schi20023970) (Table S5). The results from GO and KEGG enrichment analyses revealed significant categorization of these differentially expressed genes (DEGs) into diverse functional categories. This included lipid metabolic processes (GO:0006629), oxidoreductase activity (GO:0016491), oxidation–reduction (GO:0055114), methylation (GO:0032259), MAPK (ko04010), regulation of lipolysis in adipocytes (ko04923), calcium signaling pathways (ko04020), and biosynthesis of amino acids (ko05418) (Fig. 4B and C). There were several genes and TFs related to lipid metabolism, transcriptional activator MYB, MAPK signaling pathway, calcium signaling pathway, and nitrogen metabolism. For instance, *SlFAD2* (schi20002880), *SlglpQ* (schi20044000), *SlALDH* (schi20046420), *SlHSD17B8* (schi20051150), *SlACADS* (schi20032360), *SlSMT1* (schi20041080), *SlCAS1* (schi20029740), *SlRPS6KA* (schi20017510), *SlSTE11* (schi20023970), *SlNRT* (schi20036980), and *SlNR* (schi20036960) were up-regulated in the treatment compared with control group (Table S6). In contrast, *SlNSDHL* (schi20036690), *SlSLC27A4* (schi20055190), *SlLPCAT2* (schi20058830), *SlDGKI* (schi20024020), *SlCALM* (schi20038920), *SlCAMK1* (schi20006460), *SlMYB3R1* (schi20033350)*, SlGRE2* (schi20067700) and *SlMYB3R5* (schi20041240) were down-regulated in the treatment compared with control group, suggesting that up- and down-regulation of these genes by nitrogen-limited treatment may be the important influence genes affecting on the lipid biosynthesis (Fig. 4D; Table S7).

### Integration analysis of ATAC-seq and RNA-seq

**Fig. 5:**
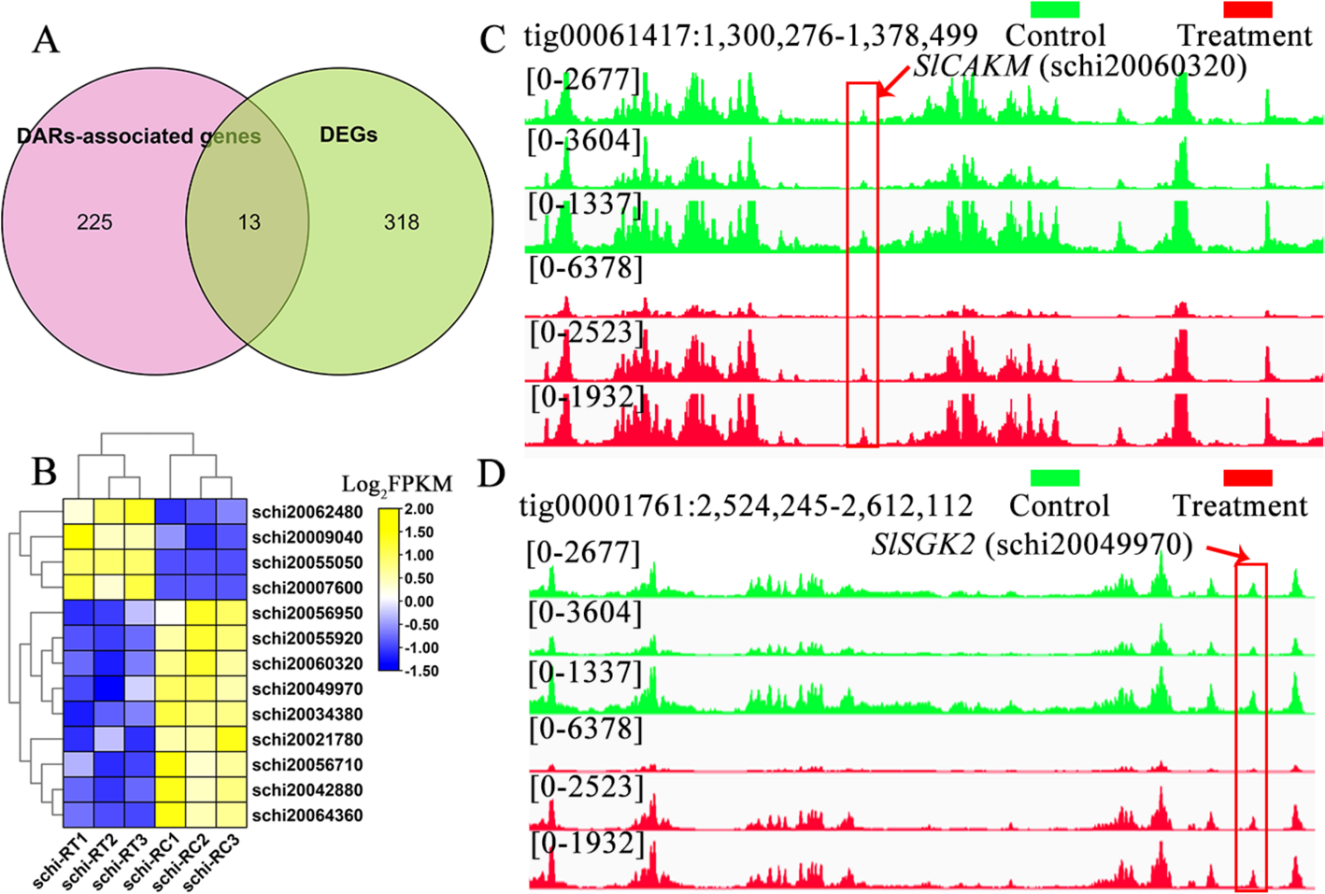
Integration analysis of ATAC-seq and RNA-seq. **A** Venn diagram of shared genes between DEGs and DARs-associated genes by RNA-seq and ATAC-seq. **B** Heatmap of 13 shared genes visualizing the changes in the expression profiles in control and treatment. **C** Overlapping analysis of *SlCAKM* gene in DEGs and DARs-associated genes by RNA-seq and ATAC-seq. **D** Overlapping analysis of *SlSGK2* gene in DEGs and DARs-associated genes by RNA-seq and ATAC-seq

By employing association analysis of differentially accessible regions (DARs) derived from ATAC-seq, we predicted that the open chromatin region caused by nitrogen-limited treatment led to changes in the ability of the promoter regulatory region, ultimately leading to the modulation of downstream gene expression, both upregulation and downregulation. A venn diagram analysis revealed that 13 genes were shared by both DEGs and DARs-associated genes, while 331 genes were unique to DEGs obtained from RNA-seq and 238 genes (327 peaks) were unique to DARs-associated genes obtained from ATAC-seq (Fig. 5A). The 13 shared genes exhibited significant expression differences, with 6 up-regulated and 7 down-regulated, and were involved in the MAPK signaling pathway, signaling and cellular processes, signal transduction, and organismal systems, including *SlCAKM* (schi20060320), *SlIFT74* (schi20034380), *SlRP2* (schi20056950), *SlSHOC2* (schi20009040), *SlTN* (schi20021780), *SlSGK2* (schi20049970), *SlHMP* (schi20062480), *SlOGT* (schi20042880), *SlclpB* (schi20064360), and *SlDNAAF3* (schi20055050) (Table S8). We analyzed and compared some of the 13 DARs-associated genes in the open chromatin region and found that they showed significant changes in the ATAC-seq data (Fig. 5B). For example, the open chromatin and the differential genes of *SlCAKM* and *SlSGK2* were suppressed by treatment medium culture relative to the control medium (Fig. 5C and D). It is likely that these genes specifically regulate the process of fatty acid and DHA biosynthesis in *S. limacinum* during nitrogen-limited treatment by a complex regulatory network of multiple metabolic pathways interacting with each other.

### Validation of the candidate genes by RT- qPCR

To further validate the gene expression levels, we selected 10 candidate genes (*SlFAD2*, *SlALDH*, *SlMYB98*, *SlMKK*, *SlCALM*, *SlCAMK1*, *SlMYB3R1*, *SlMYB3R5*, *SlSGK2*, and *SlCALM*) for qRT-PCR analysis. The results of qRT-PCR were consistent with the expression levels obtained from RNA-Seq and ATAC-seq (Figure S5).

## Discussion

To develop algae and fungus species with enhanced fatty acid and DHA levels, a deep understanding of the lipid biosynthesis regulatory mechanisms is essential. Recent research has shed light on the impact of nitrogen limitation on fatty acid and DHA biosynthesis pathways. However, the detailed molecular mechanisms governing these biosynthetic processes in response to nitrogen limitation at various culture stages are not fully understood. Previous studies have confirmed that nitrogen limitation effectively boosts fatty acid and DHA accumulation [12], but there has been limited investigation into how chromatin accessibility influences transcriptional regulation during this process. In this study, we aimed to fill this research gap by analyzing the chromatin accessibility under nitrogen-limited conditions using ATAC-seq. We identified crucial transcription factors and functional genes that play significant roles in fatty acid and DHA biosynthesis. By combining ATAC-seq and RNA-seq analyses post-nitrogen limitation treatment, we gained insights into how chromatin opening influences gene expression and, subsequently, the biosynthesis of fatty acids and DHA. This integrative approach allows us to better understand the complex interplay between chromatin structure and gene regulation, specifically under nutrient-stressed conditions, paving the way for more efficient strategies in enhancing fatty acid and DHA production in algae and fungus.

Following the completion of KEGG and GO enrichment analyses, the outcomes indicated the participation of peak-associated genes in various processes, including lipid transport and metabolism, as well as signal transduction mechanisms. MYB transcription factors, and energy production and conversion were significantly enriched, including *SlSCP2* (schi20031420), *SlSLD* (schi20021550), *SlSNQ2* (schi20046190), *SlALDH* (schi20015830), *SlMYB98* (schi20043810), and *SlMKK* (schi20040620). This indicates that nitrogen-limited treatment could play an important role in the regulation of fatty acid and DHA biosynthesis in *S. limacinum*. *SCP2*, also known as sterol carrier protein-2, plays a pivotal role in facilitating the transportation of lipids across membranes [17]. *SLD*, a delta8-fatty-acid desaturase, was initially identified in the protist Euglena gracilis. This enzyme introduces a double bond at the 8-position in 20-carbon fatty acids and plays a significant role in the synthesis of eicosanoids from precursors, including polyunsaturated fatty acids with chain lengths of 20 carbons or longer. *SLD* is a vital component of the alternative pathway for the biosynthesis of longer chain polyunsaturated fatty acids [18]. *SNQ2* is a gene encoding an ATP-binding cassette (ABC) transporter. ABC transporters play a crucial role in the transport and distribution of lipids and are closely associated with fatty acid metabolism. They have a great influence on the seed oil yield of camelina, and it has been found that ABC transporter can potentially be applied to improving oil production for *camelina* [19]. Studies have also revealed a fundamental similarity in the core biochemical function of peroxisome ABC transporters between oilseeds and cereals; however, its physiological effects and importance may vary [16]. By thoroughly characterizing the peroxisomal ABC transporter defective mutant, it showed that TAG is a key intermediate in mobilizing fatty acids from membrane lipids for peroxisome beta oxidation under prolonged dark treatment [20]. Numerous studies have established a strong connection between ABC transporters and fatty acid metabolism. Acetaldehyde dehydrogenase (*ALDH*) genes are ubiquitously found across various organisms, with *Arabidopsis* possessing a total of 14 *ALDHs*. Among these, *ALDH3I1, ALDH3H1*, and *ALDH7B4* exhibit tissue-specific activation in response to dehydration, high salinity, and ABA (abscisic acid), respectively. Transcriptional analysis of *Arabidopsis* mutants, namely ALDH3I1 and ALDH7B4, provides insights into the regulatory role of the plant hormone ABA in stress responses, as well as its impact on pathways governing glucose metabolism and the composition of membrane lipid fatty acids. ALDH genes associated with stress responses participate in diverse pathways, and their regulation encompasses various signal transduction pathways [21]. In *Arabidopsis*, the ALDH enzymes belonging to family 2 catalyze the oxidation of acetaldehyde, generating acetate for the biosynthesis of acetyl-CoA through the activity of acetyl-CoA synthetase. This metabolic pathway, known as the "pyruvate dehydrogenase bypass," is intricately connected to fatty acid biosynthesis [22]. In *S. limacinum*, we found that transcription factors MYB98 and MKK play major roles in fatty acid and DHA accumulation under nitrogen-limited treatment. Previous studies of MYB98 have mainly focused on its developmental regulatory effects on plant reproductive organs or cells of plants, such as the fertilization in flowering plants, the development of female gametophytes, and the activation of gene expression necessary for pollen tube guidance and filiform apparatus formation in synergid cells [23–25]. Our preliminary research has found that IgMYB98 may play a negative regulatory effect on fucoxanthin biosynthesis in I. galbana, a fucoxanthin-producing single-cell organism, induced by green light [26]. MKK is a crucial serine/threonine-protein kinase involved in various signaling pathways, including nitrogen signaling, among others, in eukaryotes. Additionally, MKK acts as a target for rapamycin (TOR) signaling. Research has shown that TOR plays an important role in the accumulation of triacylglycerols (TAGs), and the accumulation of lipid droplets requires de novo fatty acid synthesis, which is dependent on fatty acid synthetase. Furthermore, it has been observed that rapamycin treatment leads to an increase in triacylglycerol (TAG) content and up-regulation of DGAT gene expression in the unicellular green algae *Chlamydomonas reinhardtii*. These discoveries indicate the widespread involvement of TOR signaling in the accumulation of TAG in microalgae [27]. Some of the genes mentioned above are directly related to the synthesis of fatty acids, and some are transcription factors, all of which are involved in the regulation of substance biosynthesis. These candidate genes may therefore be involved in the biosynthesis mechanism of fatty acids and DHA caused by nitrogen-limited treatment in *S. limacinum*. By identifying and annotating 989 DARs-associated motifs, we obtained 54 target TF classes, including BPC, RAMOSA1, SPI1, MYC, and MYB families. AtBPC6 was found to regulate photosynthesis, photoreactions, photosynthetic membranes, and reproductive development during flower morphogenesis in petunias [28]. BPC1 has the ability to induce the upregulation of the *Arabidopsis thaliana* SEEDSTICK gene (STK), thereby governing the identity of ovules [29]. Additionally, BPC1 may play an important role as a calcium-sensitive signal molecule in pollination of Arabidopsis thaliana [30]. MYB3, characterized as a transcriptional repressor, plays an important role in lignin and anthocyanin biosynthesis under salt stress conditions in Arabidopsis [31]. As a positive regulator, AtMYB111 expression could be highly induced under salt treatment in Arabidopsis, and its role was dependent on its regulation of flavonoid synthesis [32]. These transcription factors primarily regulate the biosynthesis of plant substances, and further studies on their function verification should be carried out in the future to investigate their potential role in the synthesis of fatty acids and DHA.

To better understand the regulatory mechanism of fatty acid and DHA accumulation, transcriptomics was performed on *S. limacinum* under nitrogen-limited treatment, revealing candidate genes involved in fatty acid and DHA metabolism. In schi-RC compared with schi-RT, we found up-regulation of *SlFAD2*, *SlALDH*, *SlHSD17B8*, *SlglpQ*, *SlACADS*, *SlSMT1*, *SlCAS1*, *SlRPS6KA*, *SlSTE11*, *SlNRT*, and *SlNR*, while *SlNSDHL*, *SlSLC27A4*, *SlLPCAT2*, *SlDGKI*, *SlCALM*, *SlCAMK1*, *SlMYB3R1*, and *Slmyb3r5* were down-regulated. Among these genes, *SlFAD2*, *SlALDH*, *SlHSD17B8*, *SlglpQ*, *SlACADS*, *SlSMT1*, *SlCAS1*, *SlNSDHL*, *lSLC27A4*, *SlLPCAT2*, and *SlDGKI* are directly related to fatty acid biosynthesis, and *SlRPS6KA*, *SlSTE11*, *SlCALM*, *SlCAMK1*, *SlMYB3R1*, and *SlMYB3R5* are transcription factors that regulate the biosynthesis process. *SlFAD2*, which encodes omega-6 fatty acid desaturase, is positioned within the endoplasmic reticulum and plastid membrane of plant cells. Its primary role entails the insertion of a double bond between carbons 12 and 13 of the monounsaturated fatty acid oleic acid, ultimately generating a polyunsaturated fatty acid with two double bonds, namely linoleic acid [33]. It is a key enzyme in the formation of linoleic acid through oleic acid dehydrogenation and a rate-limiting enzyme in the metabolic pathway of polyunsaturated fatty acids, governing the synthesis of the majority of unsaturated fats in plant cells. Functional studies have demonstrated the significant involvement of *IgFAD2*, isolated from the marine microalgae *I. galbana*, in the biosynthetic pathways of polyunsaturated fatty acids [34]. Recently, the content and composition of unsaturated fatty acids have been shown to be regulated by mutations and modifications of the *FAD2* gene in many plants [34–37]. We found that nitrogen-limited treatment significantly up-regulated the expression of SlFAD2 gene, indicating its important role in increasing the contents of fatty acids and DHA in *S. limacinum*. In addition, *SlALDH* was also significantly up-regulated in both ATAC-seq and transcriptome differential expression gene analysis, indicating the potential role of *IgFAD2* in regulating fatty acid metabolism, potentially through the regulation of metabolic flow. Therefore, we concluded that *SlALDH* plays an important role in the effects of nitrogen-limited treatment on the contents of fatty acids and DHA in *S. limacinum*. Notably, among the identified transcription factors and regulatory genes, *SlRPS6KA*, *SlSTE11*, *SlCALM*, *SlCAMK1*, *SlMYB3R1*, and *SlMYB3R5* belong to plant MAP kinase signaling components that play crucial roles in plant growth and development. These components include the MEKK/STE11, RAF, MLK, and CDC7 families [38]. Previous studies have proposed that the *A. thaliana ATMEKK1* might be involved in the osmotic stress response pathway and could mediate the response at the level of the MEKK kinase ste11 in *Saccharomyces cerevisiae* [39]. Calmodulin (CaM) is a significant regulator that plays diverse roles in the growth and development of plants. Several studies have shown that *CALM* gene expression changes are correlated with metabolic changes of fatty acids, suggesting that *CALM* exerts a regulatory influence on the metabolism of fatty acids [40–46]. In our study, we observed a significant decrease in the expression of *SlCALM* under nitrogen stress treatment. Calmodulin kinases (CaM kinases) are serine/threonine protein kinases that are regulated by calcium/calmodulin complexes downstream of *CALM*. We also observed a significant down-regulation of *SlCAMK1* during nitrogen-limited treatment, which is consistent with the down-regulation of *SlCALM*. Additional investigation is required to elucidate the precise mechanism by which these genes operate. The MYB transcription factor subclass contains three R domains, and MYB3R proteins in plants are mainly involved in the regulation of cell cycle, cell differentiation, plant tolerance to adversity, and the synthesis of plant metabolites [47–50]. To further elucidate the regulatory dynamics of fatty acid and DHA production, we undertook transcriptomic analysis on *S. limacinum* subjected to nitrogen-deprivation. This revealed a cadre of genes integral to fatty acid and DHA metabolism. Comparing schi-RC to schi-RT, transcription factors like *SlRPS6KA, SlSTE11*, and *SlMYB3R1* potentially influence the biosynthesis procedure. In our study, we found that two MYB3R genes, *SlMYB3R1* and *SlMYB3R5*, were significantly down-regulated under nitrogen-limited treatment, which negatively regulated the synthesis of fatty acids and DHA. These genes are promising candidates for further investigation.

In our integrated analysis of DARs of ATAC-seq, we found that 13 genes were shared by DEGs and DARs-associated genes, namely *SlCAMK*, *SlIFT74*, *SlRP2*, *SlSHOC2*, *SlTN*, *SlSGK2*, *SlHMP*, *SlOGT*, *SlclpB*, *SlDNAAF3*, and *SlE2.4.1.173*. Many studies have demonstrated the close relationship between *CAMK* and *SGK2* and plant fatty acid metabolism. *CAMK1* participates in the encoding and synthesis of calcineurin, and in Arabidopsis thaliana, the calcineurin B-like (CBL) protein serves a vital function in the regulation of ion fluxes and responses to abiotic stress. *Arabidopsis* seeds with the cbl2/3rf mutation displayed reduced size, weight, and fatty acid content when compared to the wild-type seeds [51]. B-like (CBL)-interacting protein kinase 9 (*CIPK9*) of Brassica napus was reported to regulate seed oil content, and both rapeseed knock-out lines and gene-silenced lines showed decreased levels of polyunsaturated fatty acids [52]. *SGK2* is responsible for encoding a ribosomal protein S6-like protein that is localized in the plastids, and by map-based cloning, it was revealed that the *Arabidopsis* rfc (regulator of fatty acid composition) 3 gene can alter the fatty acid composition of membrane lipids, resulting in an increase in oleic acid (18:1) levels and a decrease in α-linolenic acid (18:3) levels among total fatty acids [53–55]. In our integration analysis, we found that *SlCAKM* was down-regulated and *SlSGK2* was up-regulated under nitrogen-limited treatment. It is possible that the levels of polyunsaturated fatty acids increased with *SlCAKM* down-regulation, while SlSGK2 up-regulation increased the degree of unsaturated fatty acids by enhancing the expression levels of FAD2 and FAD3 [53]. Therefore, *SlCAKM* may act as a negative regulator of fatty acid and DHA synthesis, while *SlSGK2* as a positive regulator, which requires further investigation. Our findings offer valuable insights into the molecular regulatory mechanisms of fatty acids and docosahexaenoic acid (DHA) in *S. limacinum* and may aid in the development of high content fatty acid and DHA strains for *S. limacinum*.

## Conclusions

*S. limacinum* holds significant importance for the commercial production of natural DHA. While a few studies have initially explored the transcriptional regulatory mechanisms of DHA in *S. limacinum* preliminarily, it is still unknown whether chromatin remodeling is related to DHA biosynthesis and how it responds to nitrogen limitation-mediated culture. This study analyzed the chromatin opening profile of nitrogen-limited using ATAC-seq and identified the key transcription factors and functional genes that affect fatty acid and DHA biosynthesis through ATAC-seq and RNA-seq analysis after nitrogen-limited treatment. The study found that *SlFAD2*, *SlALDH*, *SlMYB98*, *SlMKK*, *SlCALM*, *SlCAMK1*, *SlMYB3R1*, *SlMYB3R5*, *SlSGK2*, and *SlCALM* genes were significantly enriched in lipid transport and metabolism, signal transduction mechanisms, and energy production and conversion. These candidate genes may be involved in the biosynthesis mechanism of fatty acids and DHA caused by nitrogen-limited treatment in *S. limacinum*. The findings of this study will facilitate in-depth understanding the molecular regulation mechanisms of fatty acid and DHA biosynthesis in *S. limacinum* and its role in response to nitrogen limitation stress, providing theoretical basis and technical support for the construction of high content fatty acid and DHA strains.

## Materials and methods

### Samples and culture conditions

The *S. limacinum* MYA-1381 strain was procured from the American Type Culture Collection (ATCC). For the experiment, two types of mediums were prepared: the control medium contained 5.0 g glucose, 1 g peptone, and 1.0 g yeast extract, all dissolved in 1L of seawater; the treatment medium was similar, but contained only 0.5 g yeast extract. Algal liquid cultures were then inoculated at a concentration of 1% into 2L Erlenmeyer flasks, each filled with 1L of the respective medium, and incubated at 28 °C and 200 rpm for 48 h. Afterward, the algae solution was portioned into 250 mL centrifuge tubes and spun at 8,000 rpm and 20 °C for 5 min, with the supernatant subsequently removed. The collected pellet was then resuspended in 10 mL of PBS buffer within a sterile 50 mL centrifuge tube, centrifuged again under the same conditions, and the supernatant discarded. Lastly, the retrieved pellet was combined with 10 mL of sterilized ultra-pure water and centrifuged at 10,000 rpm and 20 °C for 10 min. After removing the supernatant, the resulting algae mud was promptly flash-frozen in liquid nitrogen and stored at -80 °C for subsequent fatty acid and DHA detection as well as omics research. For the purpose of examining the build-up of fatty acids and DHA within *S. limacinum*, under different nitrogen supply conditions, including nitrogen-replete (NR) as control and nitrogen-limited (NL) as treatment conditions. The incubation time was 12 h, 24 h,36 h,48 h,60 h and 72 h. We measured the content of fatty acids and DHA in control and nitrogen-limited at 6 fermentation stages of algae by GC–MS.

### Fatty acid and DHA content detection

Fifty milliliters of the algal liquid cultures were spun at 8,000 rpm for 5 min, following which the supernatant was removed. The resulting algal slurry was subsequently frozen at -80℃ and then dried in a vacuum freeze dryer for a duration of 48 h. The dry weight of this dehydrated algal slurry was then recorded.

For fatty acid extraction, 50 mg of algal powder was combined with 3 mL of a 10% NaCl solution, then thoroughly mixed using a vortex mixer until no clumps remained. Subsequently, an equal volume of a chloroform:methanol mixture (2:1 ratio) was added and vortexed, followed by centrifugation at 8,000 rpm for 5 min. The bottom layer contained the fatty acids within the chloroform phase. The middle layer was cautiously discarded, while the chloroform phase was transferred into a fresh tube. This process was carried out twice more to ensure complete collection of all chloroform phases. The chloroform solution containing fatty acids was blown dry with a nitrogen blower to obtain the fatty acid extract, which could be stored at -80 °C.

For fatty acid composition analysis, the fatty acid extract was dissolved in 1 mL of toluene. Then, 200μL of 1 mg/mL BHT antioxidant and 2 mL of chloracetyl solution were added and mixed well. The solution was incubated overnight at 50 °C. The next day, 1 mL of n-hexane containing 1 mg/mL methyl 19 acid was added to the fatty acid and mixed. After standing and stratifying, the upper n-hexane layer was passed through a 0.22 μm nylon membrane for gas chromatography analysis.

The gas chromatography used was an Agilent 7697B, with a detector set at FID and temperature maintained at 250℃. The chromatographic analysis was conducted on a SUPELCO SPTM-2560 column (100 m × 0.25 mm × 0.2 μm) with a flow rate set at 1.2 mL/min. Nitrogen served as the carrier gas and the injection volume was fixed at 1μL, with a split ratio of 10:1. The oven temperature program started at 140℃ for 5 min, followed by a ramp of 4℃/min up to 250℃, which was then maintained for 12.5 min. The blend, DHA, and EPA standards were examined, and standard curves for each fatty acid were generated using the internal standard method, facilitating the computation of each fatty acid's content in the samples under scrutiny.

### ATAC sequencing and analysis

Six samples were used for ATAC sequencing: schi-AC1, schi-AC2, schi-AC3 (control: samples cultured for 48 h on control medium), and schi-AT1, schi-AT2, and schi-AT3 (treatment: samples cultured for 48 h on treatment medium). Algal cells underwent centrifugation at 4℃ for 5 min at 500 g, following which the supernatant was discarded. The cells were then given a wash with cold PBS, with the resultant supernatant similarly removed after an additional centrifugation under identical conditions. Subsequently, the cells were resuspended in cold lysis buffer and subjected to a final centrifugation step at 4℃ for 10 min at 500 g to eliminate the supernatant. The transposition reaction was prepared using the Tn5 transposase. Nuclei were resuspended in this transposition mixture and incubated at 37℃ for 30 min prior to the DNA purification step. The purified DNA was subjected to PCR amplification reactions and the final libraries were run on the Illumina platform. Initial reads were managed using Cutadapt 1.8.3 [56] to ensure quality control. Following this, the refined reads were mapped onto the S. limacinum MYA-1381 genome via Bowtie2 (version 2.2.4) [57]. Coverage maps were subsequently generated with deepTools2.07 [58] and peak calling was done with MACS2.1.1, with a threshold of FDR < 0.05. The annotation of genome-wide peak was performed with ChIPseeker1.2.6 [59, 60]. Motif identification and annotation were executed through MEME-ChIP4.11.2 [61]. We used DiffBind2.2.11 to pinpoint differential peaks, setting parameters for a fold change of 1.2 or greater and a false discovery rate (FDR) of less than 0.05. We annotated the genes associated with the differentially accessible regions (DARs) by cross-referencing them with various databases, including NR, Swiss-prot, GO, KEGG, COG, KOG, eggNOG, and Pfam [62–68]. GO and KEGG enrichment of DARs-associated genes were analyzed using the R package clusterProfiler4.2 [64, 65].

### mRNA sequencing and analysis

Six samples were used for mRNA sequencing, labeled as schi-RC1, schi-RC2, schi-RC3 (control: samples cultured for 48 h on control medium), and schi-RT1, schi-RT2, and schi-RT3 (treatment: samples cultured for 48 h on treatment medium). The extraction of total RNA was performed using TRIzol reagent (Invitrogen Life Technologies, CA, USA). To ensure purity and quality, the extracted RNA was assessed using a NanoPhotometer (IMPLEN, CA, USA) and a 2100 Bioanalyzer (Agilent Technologies, USA). For the preparation of RNA libraries, the previously described method was employed, and subsequently, sequencing was carried out on the Novaseq 6000 platform using PE150. Trimmomatic (version 0.36) was used to obtain clean reads from Illumina raw reads, which were then mapped to the genome using HISAT2 (version 2.2.1) [69, 70]. EdgeR (version 4.2) was employed to detect differentially expressed genes (DEGs) using the thresholds of |log2 Fold change| being equal to or greater than 1.5, and a p-value being less than 0.05. For enrichment analysis, the topGO R package (version 3.8) was used for Gene Ontology (GO), while KOBAS (version 3.0) was used for KEGG pathway analysis [71].

### Combined analysis of ATAC-Seq and RNA-Seq data

The genes related to differential peaks identified by ATAC-seq were compared with the differentially expressed genes (DEGs) identified by RNA-seq. Specifically, the upregulated peaks from ATAC-seq were compared with the associated upregulated DEGs from RNA-seq, and the downregulated peaks from ATAC-seq were compared with the associated downregulated DEGs from RNA-seq. Genes identified from both ATAC-seq and RNA-seq analyses were further analyzed using Gene Ontology (GO) and KEGG enrichment, utilizing the topGO R package (version 3.8) and KOBAS (version 3.0) respectively, as outlined by Xie et al. [71].

### Real-time quantitative PCR

Total RNA extraction was carried out using the TRIzol Kit (Takara, Tokyo, Japan). Subsequently, cDNA synthesis was facilitated by the TransScript One-Step gDNA Removal and cDNA synthesis SuperMix (Beijing TransGen Biotech Co., Ltd.). RT-PCR was executed with the aid of the TransScript® Green One-Step qRT-PCR SuperMix Kit (TransGen Biotech, China), with glyceraldehyde-3-phosphate dehydrogenase (GAPDH) utilized as a reference gene.

### Supplementary Information

**Additional file 1:** **Table S1.** Evaluation statistics of sample sequencing data. **Table S2.** Mapping statistical of each sample. **Table S3.** Distribution statistics of Reads in gene functional elements. **Table S4.** Motif enrichment analysis showed the potential functions of DARs-associated genes between control and treatment group. **Table S5.** TFs identification of DEGs between control and treatment group. **Table S6.** Annotation of up-DEGs in the treatment compared with control group. **Table S7.** Annotation of down-DEGs in the treatment compared with control group. **Table S8.** Annotation of shared genes from DEGs and DARs-associated genes by RNA-seq and ATAC-seq.

**Additional file 2:** **Fig. S1.** KEGG (a) and GO (b) enrichment analysis showed the potential functions of peak-associated genes in the control group. **Fig. S2.** KEGG (a) and GO (b) enrichment analysis showed the potential functions of peak-associated genes in the treatment group. **Fig. S3.** GO enrichment in biological process (a) and molecular function (b) terms analysis showed the potential functions of DARs-associated genes between control and treatment group. **Fig. S4.** KEGG enrichment analysis showed the potential functions of DARs-associated genes between control and treatment group. **Fig. S5.** Real-time quantitative RT-qPCR confirmation of SlFAD2, SlALDH, SlMYB98, SlMKK, SlCALM, SlCAMK1, SlMYB3R1, SlMYB3R5, SlSGK2, and SlCALM. Relative gene expressions were analyzed using the 2−ΔΔCt method. Experiments were performed in triplicate. Error bars indicate standard deviation.

## Data Availability

The ATAC sequencing data have been archived at the National Genomics Data Center, Beijing Institute of Genomics, Chinese Academy of Sciences, under the BioProject accession number CRA010780 (https://ngdc.cncb.ac.cn/gsa). For annotation and biological analyses, raw RNA-seq sequencing data were utilized and are available in the BIG Subsystem with the BioProject accession number PRJCA002397 (http://bigd.big.ac.cn).
