# Supplementary material for: Integration analysis of ATAC-seq and RNA-seq provides insight into fatty acid biosynthesis in Schizochytrium limacinum under nitrogen limitation stress
Source: BMC Genomics. 2024 Feb 5;25:141. doi: 10.1186/s12864-024-10043-5 (PMC10840233; doi:10.1186/s12864-024-10043-5)
Supplement: Supplementary file 1 — Additional file 1: Table S1. Evaluation statistics of sample sequencing data. Table S2. Mapping statistical of each sample. Table S3. Distribution statistics of Reads in gene functional elements. Table S4. Motif enrichment analysis showed the potential functions of DARs-associated genes between control and treatment group. Table S5. TFs identification of DEGs between control and treatment group. Table S6. Annotation of up-DEGs in the treatment compared with control group. Table S7. Annotation of down-DEGs in the treatment compared with control group. Table S8. Annotation of shared genes from DEGs and DARs-associated genes by RNA-seq and ATAC-seq. [file 12864_2024_10043_MOESM1_ESM.docx]

Table S1 Evaluation statistics of sample sequencing data

| SampleID | Raw_Reads | Clean_Reads | GC(%) | N(%) | Q30(%) |
| --- | --- | --- | --- | --- | --- |
| schi-AC1 | 136,984,121 | 136,888,468 | 47.35 | 0 | 95.08 |
| schi-AC2 | 106,602,889 | 106,515,578 | 47.04 | 0 | 95.23 |
| schi-AC3 | 95,084,272 | 95,032,573 | 47.38 | 0 | 92.73 |
| schi-AT1 | 106,402,331 | 106,306,707 | 46.96 | 0 | 95.30 |
| schi-AT2 | 100,320,397 | 100,218,518 | 46.48 | 0 | 94.74 |
| schi-AT3 | 105,632,328 | 105,512,779 | 46.88 | 0 | 95.17 |

Note: SampleID: sample analysis number; Raw_Reads: number of Reads of the original data; Clean_Reads: number of filtered Clean Data Reads; GC(%) : GC content of Clean Data after filtration, i.e. the percentage of G and C bases in the total bases of Clean Data; N(%): N base content in Clean Data; Q30(%) : The percentage of bases with mass value greater than or equal to 30 in the filtered Clean Data.

Table S2 Mapping statistical of each sample

| Sample name | Total  Reads | Mapped  Reads | Mapped-Ratio  (%) | Uniq_Mapped Reads | Uniq_Mapped  Ratio  (%) | MT(%) | Duplication  (%) |
| --- | --- | --- | --- | --- | --- | --- | --- |
| schi-AC1 | 136,888,468 | 136,448,935 | 99.68 | 130,577,102 | 95.38 | 0 | 41.27 |
| schi-AC2 | 106,515,578 | 106,189,367 | 99.69 | 101,358,819 | 95.15 | 0 | 42.05 |
| schi-AC3 | 95,032,573 | 94,801,663 | 99.76 | 90,718,769 | 95.46 | 0 | 33.10 |
| schi-AT1 | 106,306,707 | 106,049,938 | 99.76 | 99,254,666 | 93.36 | 0 | 40.35 |
| schi-AT2 | 100,218,518 | 99,909,154 | 99.69 | 95,678,449 | 95.46 | 0 | 40.33 |
| schi-AT3 | 105,512,779 | 105,106,369 | 99.61 | 101,284,196 | 95.99 | 0 | 40.39 |

Note: Total_Reads: the number of Clean Reads, counted by single end; Mapped_Reads: The number and proportion of Clean Reads to the reference genome; Uniq_Mapped_Reads: The number and proportion of Clean Reads uniquely matched to the reference genome; MT(%) : The percentage of mapping to mitochondrial Reads in all Clean Reads; Duplication(%) : The percentage of redundant Reads in all Clean Reads.

Table S3 Distribution statistics of Reads in gene functional elements

| SampleID | Promoter (<=1kb)(%) | Promoter (1-2kb)(%) | Promoter (2-3kb)(%) | 3' UTR(%) | 5' UTR(%) | Exon(%) | Intron(%) | Distal Intergenic(%) |
| --- | --- | --- | --- | --- | --- | --- | --- | --- |
| schi-AC1 | 17.79 | 14.3 | 10.47 | 0.1 | 0.03 | 0.26 | 0.89 | 56.16 |
| schi-AC2 | 16.56 | 14.06 | 10.47 | 0.11 | 0.02 | 0.22 | 0.95 | 57.6 |
| schi-AC3 | 18.6 | 14.66 | 10.52 | 0.13 | 0.03 | 0.26 | 0.95 | 54.84 |
| schi-AT1 | 18.07 | 14.34 | 10.39 | 0.1 | 0.02 | 0.25 | 0.87 | 55.97 |
| schi-AT2 | 15.07 | 14.32 | 10.72 | 0.09 | 0.02 | 0.19 | 0.97 | 58.63 |
| schi-AT3 | 15.46 | 14.42 | 10.76 | 0.08 | 0.01 | 0.23 | 0.96 | 58.09 |

Table S4 Motif enrichment analysis showed the potential functions of DARs-associated genes between control and treatment group.

| TF | Total  peak | TF  peak | Cluster  peak | Cluster_TF  peak | TF | Total  peak | TF  peak | Cluster  peak | Cluster_TF  peak |
| --- | --- | --- | --- | --- | --- | --- | --- | --- | --- |
| Trl | 989 | 56 | 989 | 56 | AZF1 | 989 | 28 | 989 | 28 |
| BPC6 | 989 | 32 | 989 | 32 | AT5G02460 | 989 | 28 | 989 | 28 |
| SPI1 | 989 | 28 | 989 | 28 | ZNF24 | 989 | 14 | 989 | 14 |
| Adof1 | 989 | 28 | 989 | 28 | FOXB1 | 989 | 11 | 989 | 11 |
| SFL1 | 989 | 28 | 989 | 28 | RAV1 | 989 | 10 | 989 | 10 |
| dof4.2 | 989 | 28 | 989 | 28 | Ptf1a(var.2) | 989 | 10 | 989 | 10 |
| AT2G28810 | 989 | 28 | 989 | 28 | MYB111 | 989 | 9 | 989 | 9 |
| PRDM1 | 989 | 28 | 989 | 28 | SUT1 | 989 | 7 | 989 | 7 |
| blmp-1 | 989 | 28 | 989 | 28 | BHLH13 | 989 | 7 | 989 | 7 |
| AT3G45610 | 989 | 28 | 989 | 28 | ATHB7 | 989 | 7 | 989 | 7 |
| OBP3 | 989 | 28 | 989 | 28 | HES7 | 989 | 7 | 989 | 7 |
| SGR5 | 989 | 28 | 989 | 28 | AIB | 989 | 7 | 989 | 7 |
| OBP1 | 989 | 28 | 989 | 28 | ATHB-5 | 989 | 7 | 989 | 7 |
| SVP | 989 | 28 | 989 | 28 | BHLH78 | 989 | 7 | 989 | 7 |
| AT1G69570 | 989 | 28 | 989 | 28 | StBRC1 | 989 | 7 | 989 | 7 |
| BPC1 | 989 | 28 | 989 | 28 | PHO4 | 989 | 7 | 989 | 7 |
| SPIB | 989 | 28 | 989 | 28 | BHLH104 | 989 | 7 | 989 | 7 |
| COG1 | 989 | 28 | 989 | 28 | h | 989 | 7 | 989 | 7 |
| RAMOSA1 | 989 | 28 | 989 | 28 | HES1 | 989 | 7 | 989 | 7 |
| EWSR1-FLI1 | 989 | 28 | 989 | 28 | MYC2 | 989 | 7 | 989 | 7 |
| AT5G66940 | 989 | 28 | 989 | 28 | BHLH3 | 989 | 7 | 989 | 7 |
| MYB3 | 989 | 28 | 989 | 28 | TFAP2C(var.2) | 989 | 7 | 989 | 7 |
| BPC5 | 989 | 28 | 989 | 28 | daf-12 | 989 | 7 | 989 | 7 |
| MGP | 989 | 28 | 989 | 28 | MYC4 | 989 | 7 | 989 | 7 |
| SOX10 | 989 | 28 | 989 | 28 | HES5 | 989 | 7 | 989 | 7 |
| Clamp | 989 | 4 | 989 | 4 | RSC30 | 989 | 7 | 989 | 7 |
| STZ | 989 | 4 | 989 | 4 | Foxj3 | 989 | 6 | 989 | 6 |

Table S5 TFs identification of DEGs between control and treatment group.

| gene_name | TF | logFC | PValue | FDR | DE |
| --- | --- | --- | --- | --- | --- |
| schi20021090 | bZIP | -3.053217912 | 9.06E-12 | 1.20E-08 | down-regulated |
| schi20044680 | C3H | 1.068795288 | 1.31E-05 | 0.000560164 | up-regulated |
| schi20033350 | MYB | -5.26305819 | 0.000800541 | 0.009182629 | down-regulated |
| schi20041240 | MYB | -1.14604489 | 0.002534268 | 0.019582981 | down-regulated |
| schi20023970 | WRKY | 1.420443089 | 0.009197958 | 0.046945696 | up-regulated |

Table S6 Annotation of up-DEGs in the treatment compared with control group.

| gene_id | predicted_  gene_name | KEGG_KOs | eggNOG annot |
| --- | --- | --- | --- |
| schi20036980 | NRT2 | K02575 | Nitrate transporter |
| schi20041080 | ERG6 | K00559,K08242 | Sterol 24-c-methyltransferase |
| schi20063190 | RETSAT | K09516,K15745 | Retinol saturase (All-trans-retinol 13,14-reductase) |
| schi20002560 |  |  | JmjC domain, hydroxylase |
| schi20033600 | NSUN2 | K15334,K15335 | NOP2 Sun RNA methyltransferase family, member 2 |
| schi20038210 | GS2 | K01915 | glutamine synthetase |
| schi20054450 |  |  | Zeta toxin |
| schi20041340 | CYP51A1 | K05917 | Cytochrome p450 |
| schi20057370 |  |  | ZIP Zinc transporter |
| schi20033790 |  |  | domain protein |
| schi20023290 | NEMA | K10680,K15552 | NADH flavin oxidoreductase NADH oxidase |
| schi20036960 |  | K10534 | Nitrate reductase is a key enzyme involved in the first step of nitrate assimilation in plants, fungi and bacteria (By similarity) |
| schi20053670 | TWISTNB | K03004 | Twist neighbor |
| schi20002880 |  | K10256,K10257,K12418 | linoleoyl-CoA desaturase activity |
| schi20006660 | RPS28 | K02979 | 40s ribosomal protein |
| schi20042040 |  |  | Oxidoreductase family, C-terminal alpha/beta domain |
| schi20062480 | HMP | K05916,K07006 | Is involved in NO detoxification in an aerobic process, termed nitric oxide dioxygenase (NOD) reaction that utilizes O(2) and NAD(P)H to convert NO to nitrate, which protects the bacterium from various noxious nitrogen compounds. Therefore, plays a central role in the inducible response to nitrosative stress (By similarity) |
| schi20038890 | AMT | K03320 | ammonium transporter |
| schi20041050 | PGUG_00946 | K07566 | yrdC domain containing (E. coli) |
| schi20023300 | SKP1 | K03094 | ubiquitin-dependent protein catabolic process |
| schi20023770 |  |  | Methyltransferase-like protein |
| schi20044680 |  | K11294 | RNA binding protein |
| schi20062500 |  |  | Putative methyltransferase |
| schi20054880 |  | K03305 | transporter |
| schi20033220 | KLC3 | K10407 | Kinesin light chain |
| schi20054870 |  | K03305 | transporter |
| schi20032360 | MT1283 | K00248 | Dehydrogenase |
| schi20023760 |  | K11294,K14411 | RNA recognition motif. (a.k.a. RRM, RBD, or RNP domain) |
| schi20017280 | OSI_08040 | K00550 |  |
| schi20013480 | PRIM2 | K02685 | DNA primase large |
| schi20037380 | LYAR | K14785,K15263 | Ly1 antibody reactive homolog (mouse) |
| schi20025280 | CYP | K01802,K03767 | PPIases accelerate the folding of proteins (By similarity) |
| schi20025970 | MAK16 | K14831 | Mak16 protein C-terminal region |
| schi20028460 | NOP10 | K11130 | H ACA ribonucleoprotein complex subunit 3 |
| schi20053870 | GINS1 | K10732 | DNA replication complex GINS protein |
| schi20065720 | YCHM | K03321 | Sulfate transporter |
| schi20034390 | NSUN5 | K15264 | NOP2 Sun domain family member |
| schi20013040 | PYROXD2 |  | pyridine nucleotide-disulphide oxidoreductase domain 2 |
| schi20038820 |  | K10357 | MYSc |
| schi20037050 | NDOR1 | K14561 | FAD binding domain |
| schi20063810 | BCCIP | K15262 | Involved in nuclear export, actin cytoskeleton organization and vesicular transport (By similarity) |
| schi20000490 |  |  | Oxidoreductase family, NAD-binding Rossmann fold |
| schi20046420 | MSC7 | K00128 | Aldehyde dehydrogenase |
| schi20029740 | CAS1 | K01853,K06045 | synthase |
| schi20028380 |  |  | Tetratricopeptide repeat |
| schi20009040 | LRRC69 | K19613 | leucine rich repeat |
| schi20042360 | NUDT5 | K01515,K13987 | nudix (nucleoside diphosphate linked moiety X)-type motif 5 |
| schi20004480 |  |  | alcohol dehydrogenase |
| schi20027700 |  |  | monooxygenase |
| schi20055050 | DNAAF3 | K19752 | motile cilium assembly |
| schi20028140 |  |  | Leucine-rich repeat-containing protein |
| schi20044000 | GLPQ | K01126 | glycerophosphoryl diester phosphodiesterase |
| schi20064830 | HMGCS1 | K01641 | synthase |
| schi20051150 | HSD17B8 | K13370 | hydroxysteroid (17-beta) dehydrogenase 8 |
| schi20014670 | YNIC | K01838,K19270 | HAD-superfamily hydrolase subfamily IA variant 3 |
| schi20017510 | NLRX1 | K04373,K12653,K14972 | leucine rich repeat containing |
| schi20023970 |  | K11228 | Mitogen-activated protein kinase kinase kinase |
| schi20007600 |  | K05841 | glycosyl transferase family |

Table S7 Annotation of down-DEGs in the treatment compared with control group.

| gene_id | predicted_  gene_name | KEGG_KOs | eggNOG annot |
| --- | --- | --- | --- |
| schi20017360 |  |  | F-box and leucine-rich repeat protein |
| schi20021090 | SPATA4 |  | Spermatogenesis associated 4 |
| schi20026340 |  |  | Calpain-like cysteine peptidase |
| schi20040730 | ENSG00000223519 | K10392,K17914 | Kinesin family member |
| schi20026240 |  | K07376 | cgmp-dependent protein kinase |
| schi20036830 | APRA | K08651,K14645,K17734 | peptidase S8 S53 subtilisin kexin sedolisin |
| schi20028850 |  |  | Transmembrane protein 16.3 |
| schi20056950 |  | K18272 | Tubulin folding cofactor C |
| schi20027860 |  | K13750 | Sodium/calcium exchanger protein |
| schi20026820 | USP21 | K11833,K11839 | ubiquitin carboxyl-terminal hydrolase |
| schi20052490 | LAMA3 |  | LPXTG-motif cell wall anchor domain protein |
| schi20016180 | CCDC151 |  | Coiled-coil domain containing 151 |
| schi20062540 | VBP1 | K06867 | Prefoldin subunit |
| schi20059260 | OSCP1 |  | organic solute carrier partner 1 |
| schi20036690 | NSDHL | K07748,K12767 | Dehydrogenase |
| schi20030820 |  |  | SAM domain (Sterile alpha motif) |
| schi20050930 | RAB23 | K06234 | RAB23, member RAS oncogene family |
| schi20015180 |  | K08332 | Armadillo beta-catenin repeat family protein |
| schi20036800 |  | K07407 | Alpha-galactosidase |
| schi20022090 |  |  | BCCT family transporter |
| schi20014890 |  | K05642,K05643,K05644,K05645,K05648 | ATP-binding cassette sub-family A ABC1 member |
| schi20007810 | IFT81 | K19677 | intraflagellar transport 81 homolog (Chlamydomonas) |
| schi20041120 | KIF13B | K17914 | kinesin family member 13B |
| schi20037590 | ARMC6 |  | ARM |
| schi20003630 |  | K06653,K12271 | Ankyrin Repeat |
| schi20018560 |  | K08857 | serine threonine-protein kinase |
| schi20042010 |  |  | Tetratricopeptide repeat |
| schi20056930 | RIB72 |  | Repeat of unknown function (DUF1126) |
| schi20002650 | TRPT1 | K04417,K04424,K10669,K18418 | tRNA phosphotransferase 1 |
| schi20001800 | EFHC2 |  | EF-hand domain (C-terminal) containing |
| schi20000360 |  |  | Kinesin family member |
| schi20039740 |  |  | Hemolysin-type calcium-binding repeat (2 copies) |
| schi20028840 |  | K01120,K13293 | Phosphodiesterase |
| schi20004470 | CCDC135 | K18402 | Coiled-coil domain containing 135 |
| schi20053470 | DNAH1 | K10408 | heavy chain |
| schi20033080 |  |  | Zinc finger, C2H2 type |
| schi20053390 | IFT172 | K19676 | intraflagellar transport 172 homolog (Chlamydomonas) |
| schi20011020 | RPS6KA2 | K04373 | ribosomal protein S6 kinase, 90kDa, polypeptide 2 |
| schi20044510 |  | K04534 | Guanine nucleotide-binding proteins (G proteins) are involved as modulators or transducers in various transmembrane signaling systems |
| schi20055960 |  |  | KISc |
| schi20056710 |  |  | Involved in proper cytoplasmic distribution of mitochondria |
| schi20033040 | ADCY10 | K11265 | adenylate cyclase 10 (soluble) |
| schi20063780 | CCDC164 | K19754 | coiled-coil domain containing 164 |
| schi20042080 |  |  | leucine rich repeat |
| schi20006320 |  | K19603 | Mitogen-activated protein kinase |
| schi20055090 |  | K04874 | potassium voltage-gated channel shaker-related subfamily member |
| schi20062320 |  | K05869 | calcium-dependent protein kinase |
| schi20000880 |  |  | ARM |
| schi20033800 | ATAT1 | K19573 | Specifically acetylates 'Lys-40' in alpha-tubulin on the lumenal side of microtubules. May affect microtubule stability and regulate microtubule dynamics |
| schi20021390 | IQCH |  | IQ motif containing H |
| schi20056690 | IFT52 | K16768,K19681 | Intraflagellar transport 52 homolog |
| schi20015940 | DAL2 | K01477 | Allantoicase |
| schi20035010 |  |  | Pfam:K_tetra |
| schi20053400 | CCDC77 | K16757 | Coiled-coil domain containing 77 |
| schi20064360 | HSP101 | K03695 | heat shock protein |
| schi20022270 |  | K18598 | Echinoderm microtubule associated protein like |
| schi20017990 |  |  | leucine Rich Repeat |
| schi20066320 | ADHA | K13979 | alcohol dehydrogenase |
| schi20024170 |  |  | Tryp_SPc |
| schi20030760 | NLRC3 |  | NLR family, CARD domain containing 3 |
| schi20067030 | KCNH7 | K04905,K04909,K04910 | Potassium voltage-gated channel, subfamily H (Eag-related), member |
| schi20038920 | CALMODULIN | K02183 | calmodulin |
| schi20000380 |  |  | guanine nucleotide exchange factor |
| schi20064840 |  | K05724 | FYVE, RhoGEF and PH domain containing |
| schi20007320 |  |  | Inherit from veNOG: primary ciliary dyskinesia protein |
| schi20067590 | ENSG00000187695 |  |  |
| schi20047200 | PPOX | K00231 | protoporphyrinogen oxidase |
| schi20013190 |  |  | WD40 |
| schi20061900 |  |  | NACHT and WD repeat |
| schi20038530 |  | K08762 | acyl-coa binding |
| schi20001310 |  |  | cell division cycle protein 48 |
| schi20035400 | PDE4D | K11600,K13293 | Phosphodiesterase |
| schi20064170 | RSPH3 |  | radial spoke 3 homolog (Chlamydomonas) |
| schi20046360 | OSM-5 | K16474 | Intraflagellar transport 88 homolog |
| schi20034170 | NLRC3 |  | NLR family, CARD domain containing 3 |
| schi20024080 |  | K06685 | Mps one binder kinase activator-like |
| schi20045570 |  | K10358 | MYSc |
| schi20049190 |  |  | Inherit from COG: Histidine kinase |
| schi20010830 |  |  | Spondin_N |
| schi20026540 |  | K11493 | regulator of chromosome condensation |
| schi20068140 |  |  | T-complex protein 10 C-terminus |
| schi20026060 |  |  | regulator of chromosome condensation |
| schi20025690 | ADCY10 | K11265 | adenylate cyclase 10 (soluble) |
| schi20055250 |  | K04615 | Gamma-aminobutyric acid B receptor |
| schi20011490 | FG04351.1 | K03013,K15361 | WD repeat domain 48 |
| schi20054340 | NEK3 | K08857 | NIMA-related kinase |
| schi20053080 | RSPH4A | K19756 | cilium axoneme assembly |
| schi20039170 | TNR | K06252 | WNT inhibitory factor 1 |
| schi20028070 |  | K10394 | Kinesin family member |
| schi20010240 | TNR | K06252 | WNT inhibitory factor 1 |
| schi20015500 | PDED |  | Lactamase_B |
| schi20030850 | NLRC3 |  | NLR family, CARD domain containing 3 |
| schi20034680 | TMEM231 | K19362 | Transmembrane component of the tectonic-like complex, a complex localized at the transition zone of primary cilia and acting as a barrier that prevents diffusion of transmembrane proteins between the cilia and plasma membranes. Required for ciliogenesis and sonic hedgehog SHH signaling |
| schi20042880 | FGSG_01916 | K09667 | Tetratricopeptide repeat |
| schi20048730 |  |  | regulator of chromosome condensation |
| schi20057010 |  |  | calcineurin |
| schi20064180 |  |  | WD domain, G-beta repeat |
| schi20009820 | PGUG_01817 | K04739,K07376,K08597 | Lactamase_B |
| schi20066070 | PTC2 | K14803 | Protein phosphatase 2C homolog |
| schi20067150 | SRP1 |  | importin subunit alpha |
| schi20052060 | PRKX | K04345,K08282,K19584 | CAMP-dependent protein kinase, catalytic subunit |
| schi20060320 |  | K02183,K06268 | calcineurin |
| schi20062120 | ARSB | K01130,K01135,K12375 | Arylsulfatase |
| schi20030740 | NLRC3 |  | NLR family, CARD domain containing 3 |
| schi20010960 | SLC24A3 | K13751,K13752 | Solute carrier family 24 (Sodium potassium calcium exchanger), member |
| schi20000940 | PDED |  | Lactamase_B |
| schi20005880 | MKK5 | K04368,K08332 | Vacuolar protein |
| schi20040170 | AKD1 | K00939,K13800,K18533 | adenylate kinase |
| schi20048540 | CGR-1 | K12850 | Transfer protein |
| schi20042100 | CCDC104 |  | Coiled-coil domain containing 104 |
| schi20060600 |  | K00870 | protein kinase kinase kinase |
| schi20033580 | WDR52 |  | WD repeat domain 52 |
| schi20063410 |  |  | whole genome shotgun sequence |
| schi20049350 |  | K10358 | myosin, heavy chain |
| schi20061080 | BRIP1 | K11136,K15362 | HELICc2 |
| schi20059770 |  |  | calcium-dependent protein kinase |
| schi20045190 |  |  | Calcium binding protein |
| schi20056640 |  | K10406 | CH |
| schi20008410 |  | K17505 | PHOsphatase |
| schi20030670 | MYO1B | K10356 | myosin IB |
| schi20008270 |  |  | Potassium voltage-gated channel, subfamily H (Eag-related), member |
| schi20027280 |  |  | Domain of unknown function (DUF389) |
| schi20050680 | TEP1 | K11127 | NACHT and WD repeat domain containing 1 |
| schi20055350 |  |  | amp-dependent synthetase and ligase |
| schi20061380 |  | K14966 | Kelch domain containing |
| schi20010870 | STK32A | K04688,K08793,K13303,K19800 | ribosomal protein s6 kinase |
| schi20034450 | S6KII | K04373 | ribosomal protein S6 kinase |
| schi20006460 | CAMK1 | K08794,K08795 | Calcium calmodulin-dependent protein kinase |
| schi20052770 | YCF45 |  | AAA |
| schi20065690 |  |  | Protein of unknown function (DUF1336) |
| schi20063850 |  |  | NACHT domain |
| schi20067800 | NLRC3 |  | NLR family, CARD domain containing 3 |
| schi20028820 | VASH1 |  | vasohibin 1 |
| schi20029900 |  | K19398 | bardet-biedl syndrome 9 |
| schi20033350 | MYB3R1 | K09420,K09422 | Transcription factor |
| schi20026770 | KIF19 | K10401 | Kinesin family |
| schi20049240 |  |  | Voltagegated Ion Channel (VIC) Superfamily |
| schi20029320 | WDR19 | K19671 | WD repeat domain 19 |
| schi20027430 | KLP2 | K10405,K10406 | Kinesin family member |
| schi20050730 |  | K01312 | peptidase, s1a (chymotrypsin) subfamily |
| schi20058170 |  | K04678,K10591,K13305 | E3 ubiquitin- protein ligase |
| schi20014820 |  | K12035 | NHL repeat |
| schi20033830 | CCDC63 |  | coiledcoil domain containing |
| schi20022370 | SPEF2 |  | nucleobase-containing compound kinase activity |
| schi20050490 |  | K02183,K13974 | Calcium binding protein |
| schi20028060 | ABAT | K01099,K13524,K14005 | Aminotransferase class-III |
| schi20009340 |  |  | MORN repeat containing |
| schi20045110 | CEP78 | K16765 | leucine Rich Repeat |
| schi20063560 | HMOX1 | K00510 | heme oxygenase (decycling) 1 |
| schi20040540 |  | K01120,K01768 | Adenylate Guanylate cyclase |
| schi20031970 |  |  | Coiled-coil domain-containing protein |
| schi20052260 | FRPC |  | Hemolysin-type calcium-binding repeat |
| schi20057080 |  |  | OTU-like cysteine protease |
| schi20006700 |  |  | to ankyrin 2,3 unc44, partial Hydra magnipapillata |
| schi20032290 |  | K10359 | myosin VIIA |
| schi20015260 |  | K17550,K19753 | leucine rich repeat |
| schi20028040 | TULP1 | K19600 | Tubby like protein 1 |
| schi20044340 |  |  | Potassium large conductance calcium-activated channel, subfamily M, alpha member 1 |
| schi20005280 | TMEM145 |  | Transmembrane protein 145 |
| schi20015200 | PDE-4 | K13293 | 3'5'-cyclic nucleotide phosphodiesterase |
| schi20015790 |  | K19043 | Peptidyl-prolyl cis-trans isomerase |
| schi20047900 |  |  | EF hand family protein |
| schi20067700 | GRE2 | K17741 | NADPH-dependent methylglyoxal reductase GRE2 |
| schi20030200 |  |  | chromosome 17 open reading frame 105 |
| schi20055190 | SLC27A4 | K08745 | solute carrier family 27 (fatty acid transporter), member 4 |
| schi20051160 |  | K14963 | wd repeat |
| schi20014340 |  | K11494,K19607 | regulator of chromosome condensation |
| schi20004080 | GPAF |  | Guanine nucleotide binding protein alpha |
| schi20035110 |  | K13752,K13753 | Solute carrier family 24 (Sodium potassium calcium exchanger), member |
| schi20010680 | PPIL6 | K12739 | PPIases accelerate the folding of proteins. It catalyzes the cis-trans isomerization of proline imidic peptide bonds in oligopeptides |
| schi20049970 |  | K13303 | Ribosomal protein S6 |
| schi20003700 | SLC34A1 | K14683 | solute carrier family 34 (sodium phosphate), member |
| schi20054930 | ANKRD39 |  | ankyrin repeat domain 39 |
| schi20041240 | MYB3R-5 | K09420,K09422 | myb domain protein 3R-5 |
| schi20017190 |  | K10357 | myosin-J heavy chain-like |
| schi20039300 |  |  | Ankyrin Repeat |
| schi20041630 |  |  | Potassium voltage-gated channel, subfamily H (Eag-related), member |
| schi20005270 | PRKG1 | K07376 | cgmp-dependent protein kinase |
| schi20022760 | WDR65 |  | WD repeat domain 65 |
| schi20059780 | SEC16B |  | SEC16 homolog B (S. cerevisiae) |
| schi20045150 | SCN2A | K04833,K04834,K04836,K04837,K04843,K16897 | Sodium channel voltage-gated type |
| schi20008820 | B9D1 | K16744 | cilium morphogenesis |
| schi20035920 |  |  | cgmp-dependent protein kinase |
| schi20065880 | KIF12 | K03178,K10399 | Kinesin family member |
| schi20043380 | CCDC176 |  | coiled-coil domain containing 176 |
| schi20027080 |  | K06515,K15377 | Solute carrier family 44 member |
| schi20003960 |  |  | Secreted subtilisin-like serine protease with keratinolytic activity that contributes to pathogenicity (By similarity) |
| schi20019150 |  | K10408 | heavy chain |
| schi20048180 | INPP5E | K01099,K01106,K03084 | inositol |
| schi20031630 | CCDC151 |  | Coiled-coil domain containing 151 |
| schi20015860 | CCDC19 |  | coiled-coil domain containing 19 |
| schi20039970 |  | K16603 | Tubulin tyrosine ligase-like family, member |
| schi20036600 |  | K13412 | calcium and calcium calmodulin-dependent serine |
| schi20010140 | KCBP |  | Kinesin family member |
| schi20027800 | DNAH1 | K10408 | heavy chain |
| schi20056620 | C15ORF26 |  | Inherit from spriNOG: chromosome 15 open reading frame 26 |
| schi20036150 | ANK2 | K06867 | ANK |
| schi20058830 | LPCAT2 | K13510 | Lysophosphatidylcholine acyltransferase |
| schi20053070 | AK7 | K00939 | Adenylate kinase 7 |
| schi20038020 | NIPAL3 |  | NIPA-like domain containing |
| schi20049750 | LRRC48 | K19753 | leucine rich repeat |
| schi20012380 |  |  | WD domain, G-beta repeat |
| schi20010490 | CCDC40 |  | coiled-coil domain containing 40 |
| schi20058740 | SLC25A2 | K15101 | Solute carrier family 25 (Mitochondrial carrier |
| schi20041960 | NLRC3 |  | NLR family, CARD domain containing 3 |
| schi20017040 | CCDC65 |  | coiled-coil domain containing 65 |
| schi20034380 | IFT74 | K19679 | Intraflagellar transport 74 homolog (Chlamydomonas) |
| schi20014770 | FCABP |  | Flagellar calcium-binding protein |
| schi20065240 | ZCF125 | K11498 | KISc |
| schi20024020 | DGKI | K00901 | diacylglycerol kinase |
| schi20004010 | APRX | K13276,K14645,K17734 | serine protease |
| schi20068110 | AMZ1 | K06974 | archaelysin family metallopeptidase |
| schi20026320 | INPP5D | K03084,K15909 | inositol polyphosphate phosphatase-like 1 |
| schi20043100 |  |  | protein Hydra magnipapillata |
| schi20012750 |  |  | Paraquat-inducible protein A |
| schi20068150 | CALM2 | K02183 | Calcium binding protein |
| schi20017530 | NLRC3 |  | NLR family, CARD domain containing 3 |
| schi20056570 | DEG7 |  | Nuclear serine protease which mediates apoptosis (By similarity) |
| schi20056380 |  | K07951 | ADP-ribosylation |
| schi20033380 | CBP |  | FG-GAP repeat |
| schi20027140 |  |  | Pfam:DUF297 |
| schi20050410 |  | K07376 | cgmp-dependent protein kinase |
| schi20050510 | IFT140 | K19672 | Intraflagellar transport 140 |
| schi20032020 | CUL1 | K03347,K03943 | CULLIN |
| schi20050700 | HIR1 |  | SPFH domain / Band 7 family |
| schi20004030 |  |  | Secreted subtilisin-like serine protease with keratinolytic activity that contributes to pathogenicity (By similarity) |
| schi20017660 | SPAG6 |  | importin subunit alpha |
| schi20032680 | PKHD1L1 | K19865 | polycystic kidney and hepatic disease 1 (autosomal |
| schi20021780 | TNR | K06252 | WNT inhibitory factor 1 |
| schi20053450 |  |  | whole genome shotgun sequence |

Table S8 Annotation of shared genes from DEGs and DARs-associated genes by RNA-seq and ATAC-seq

| ID | name | GO_terms | KOs | eggNOG annot |
| --- | --- | --- | --- | --- |
| schi20060320 |  | GO:0000003,GO:0000910,GO:0003006,GO:0003674,GO:0003824,GO:0005488,GO:0005509,GO:0005575,GO:0005622,GO:0005623,GO:0005856,GO:0006139,GO:0006152,GO:0006163,GO:0006195,GO:0006200,GO:0006725,GO:0006753,GO:0006793,GO:0006796,GO:0006807,GO:0006928,GO:0006950,GO:0007049,GO:0007275,GO:0008150,GO:0008152,GO:0009056,GO:0009116,GO:0009117,GO:0009119,GO:0009123,GO:0009125,GO:0009126,GO:0009128,GO:0009141,GO:0009143 | K02183,K06268  CALM Lipid and atherosclerosis 09130 Environmental Information Processing  09132 Signal transduction  04016 MAPK signaling pathway - plant | calcineurin |
| schi20034380 | IFT74 | GO:0003008,GO:0003674,GO:0003682,GO:0005488,GO:0005515,GO:0005575,GO:0005622,GO:0005623,GO:0005634,GO:0005737,GO:0005813,GO:0005815,GO:0005856,GO:0005929,GO:0005930,GO:0006355,GO:0006357,GO:0006810,GO:0006928,GO:0006935,GO:0007017,GO:0007018,GO:0007600,GO:0008150,GO:0009605,GO:0009889,GO:0009891,GO:0009893,GO:0009987,GO:0010468,GO:0010556,GO:0010557,GO:0010604,GO:0010628,GO:0010970,GO:0015630,GO:0016023,GO:0016043,GO:0019219,GO:0019222,GO:0030030,GO:0030155,GO:0030705,GO:0030990,GO:0030992,GO:0031323,GO:0031325,GO:0031326,GO:0031328,GO:0031410,GO:0031514 | K19679 IFT74 intraflagellar transport protein 74 | Intraflagellar transport 74 homolog (Chlamydomonas) |
| schi20056950 |  |  | K18272 | Tubulin folding cofactor C |
| schi20009040 | LRRC69 |  | K19613 SHOC2  map04014 Ras signaling pathway | leucine rich repeat |
| schi20021780 | TNR | GO:0000278,GO:0000281,GO:0000902,GO:0000910,GO:0005575,GO:0005576,GO:0005615,GO:0005622,GO:0005623,GO:0005737,GO:0005856,GO:0005886,GO:0005938,GO:0006810,GO:0006897,GO:0006909,GO:0006996,GO:0007010,GO:0007015,GO:0007049,GO:0007155,GO:0008104,GO:0008150,GO:0009653,GO:0009987,GO:0010468,GO:0010608,GO:0015629,GO:0016020,GO:0016043,GO:0016192,GO:0019222,GO:0022402,GO:0022610,GO:0030029,GO:0030036,GO:0031589,GO:0031647,GO:0032502,GO:0032989,GO:0033036,GO:0034394,GO:0034613,GO:0043226,GO:0043228,GO:0043229,GO:0043232,GO:0043933,GO:0044421,GO:0044424,GO:0044444 | K06252 | WNT inhibitory factor 1 |
| schi20056710 |  |  |  | Involved in proper cytoplasmic distribution of mitochondria (By similarity) |
| schi20049970 |  |  | K13303 | Ribosomal protein S6 |
| schi20062480 | HMP | GO:0003674,GO:0003824,GO:0006807,GO:0008150,GO:0008152,GO:0008941,GO:0016491,GO:0016651,GO:0016705,GO:0016708,GO:0044710,GO:0051213,GO:0055114 | K05916,K07006 | Is involved in NO detoxification in an aerobic process, termed nitric oxide dioxygenase (NOD) reaction that utilizes O(2) and NAD(P)H to convert NO to nitrate, which protects the bacterium from various noxious nitrogen compounds. Therefore, plays a central role in the inducible response to nitrosative stress (By similarity) |
| schi20042880 | FGSG_01916 | GO:0003674,GO:0003824,GO:0005975,GO:0006464,GO:0006486,GO:0006493,GO:0008150,GO:0008152,GO:0009058,GO:0009059,GO:0009100,GO:0009101,GO:0009987,GO:0016740,GO:0016757,GO:0019538,GO:0034645,GO:0036211,GO:0043170,GO:0043412,GO:0043413,GO:0044237,GO:0044238,GO:0044249 | K09667  OGT  protein O-GlcNAc transferase [EC:2.4.1.255] | Tetratricopeptide repeat |
| schi20064360 | HSP101 | GO:0000302,GO:0005575,GO:0005622,GO:0005623,GO:0005634,GO:0005737,GO:0005829,GO:0006950,GO:0006979,GO:0008150,GO:0008152,GO:0009266,GO:0009314,GO:0009408,GO:0009416,GO:0009507,GO:0009526,GO:0009532,GO:0009536,GO:0009570,GO:0009628,GO:0009642,GO:0009644,GO:0009941,GO:0009987,GO:0010035,GO:0019538,GO:0031967,GO:0031975,GO:0033554,GO:0034605,GO:0042221,GO:0042542,GO:0043170,GO:0043226,GO:0043227 | K03695 | heat shock protein |
| schi20055050 | DNAAF3 | GO:0000902,GO:0003341,GO:0005575,GO:0005622,GO:0005623,GO:0005737,GO:0006461,GO:0006928,GO:0006996,GO:0007017,GO:0007018,GO:0008150,GO:0009653,GO:0009987,GO:0010927,GO:0016043,GO:0022607,GO:0030030,GO:0030031,GO:0032502,GO:0032989,GO:0032990,GO:0034622,GO:0042384,GO:0043623,GO:0043933,GO:0044085,GO:0044424,GO:0044458,GO:0044464,GO:0044699,GO:0044763,GO:0044767,GO:0044782,GO:0048646,GO:0048856,GO:0048858,GO:0048869,GO:0060271,GO:0065003,GO:0070271,GO:0070286,GO:0070925,GO:0071822,GO:0071840 | K19752  DNAAF3  dynein axonemal assembly factor 3 | motile cilium assembly |
| schi20007600 |  |  | K05841 E2.4.1.173  sterol 3beta-glucosyltransferase | glycosyl transferase family |
