# Supplementary material for: Integration analysis of ATAC-seq and RNA-seq provides insight into fatty acid biosynthesis in Schizochytrium limacinum under nitrogen limitation stress
Source: BMC Genomics. 2024 Feb 5;25:141. doi: 10.1186/s12864-024-10043-5 (PMC10840233; doi:10.1186/s12864-024-10043-5)
Supplement: Supplementary file 2 — Additional file 2: Fig. S1. KEGG (a) and GO (b) enrichment analysis showed the potential functions of peak-associated genes in the control group. Fig. S2. KEGG (a) and GO (b) enrichment analysis showed the potential functions of peak-associated genes in the treatment group. Fig. S3. GO enrichment in biological process (a) and molecular function (b) terms analysis showed the potential functions of DARs-associated genes between control and treatment group. Fig. S4. KEGG enrichment analysis showed the potential functions of DARs-associated genes between control and treatment group. Fig. S5. Real-time quantitative RT-qPCR confirmation of SlFAD2, SlALDH, SlMYB98, SlMKK, SlCALM, SlCAMK1, SlMYB3R1, SlMYB3R5, SlSGK2, and SlCALM. Relative gene expressions were analyzed using the 2−ΔΔCt method. Experiments were performed in triplicate. Error bars indicate standard deviation. [file 12864_2024_10043_MOESM2_ESM.doc]

a


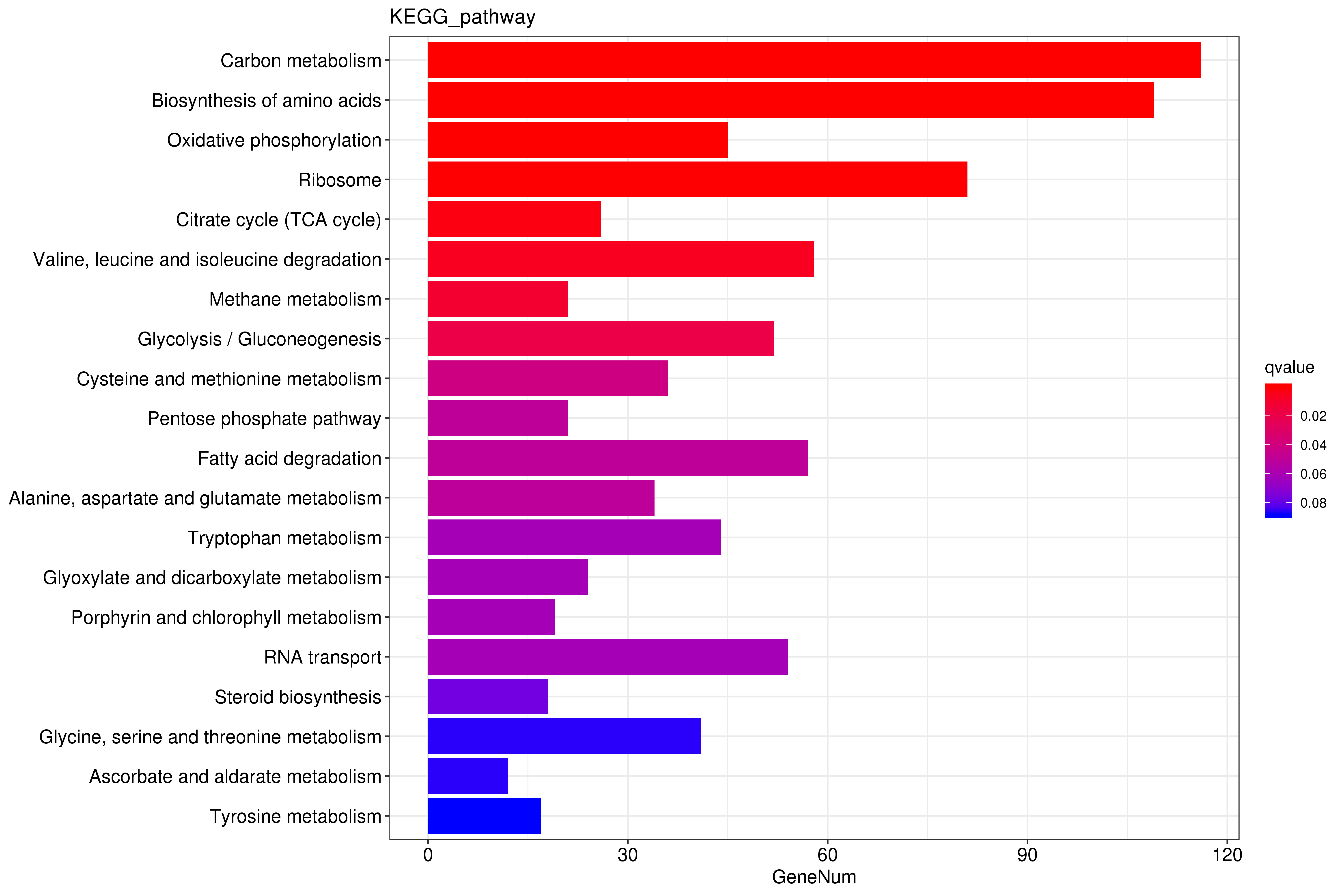


b


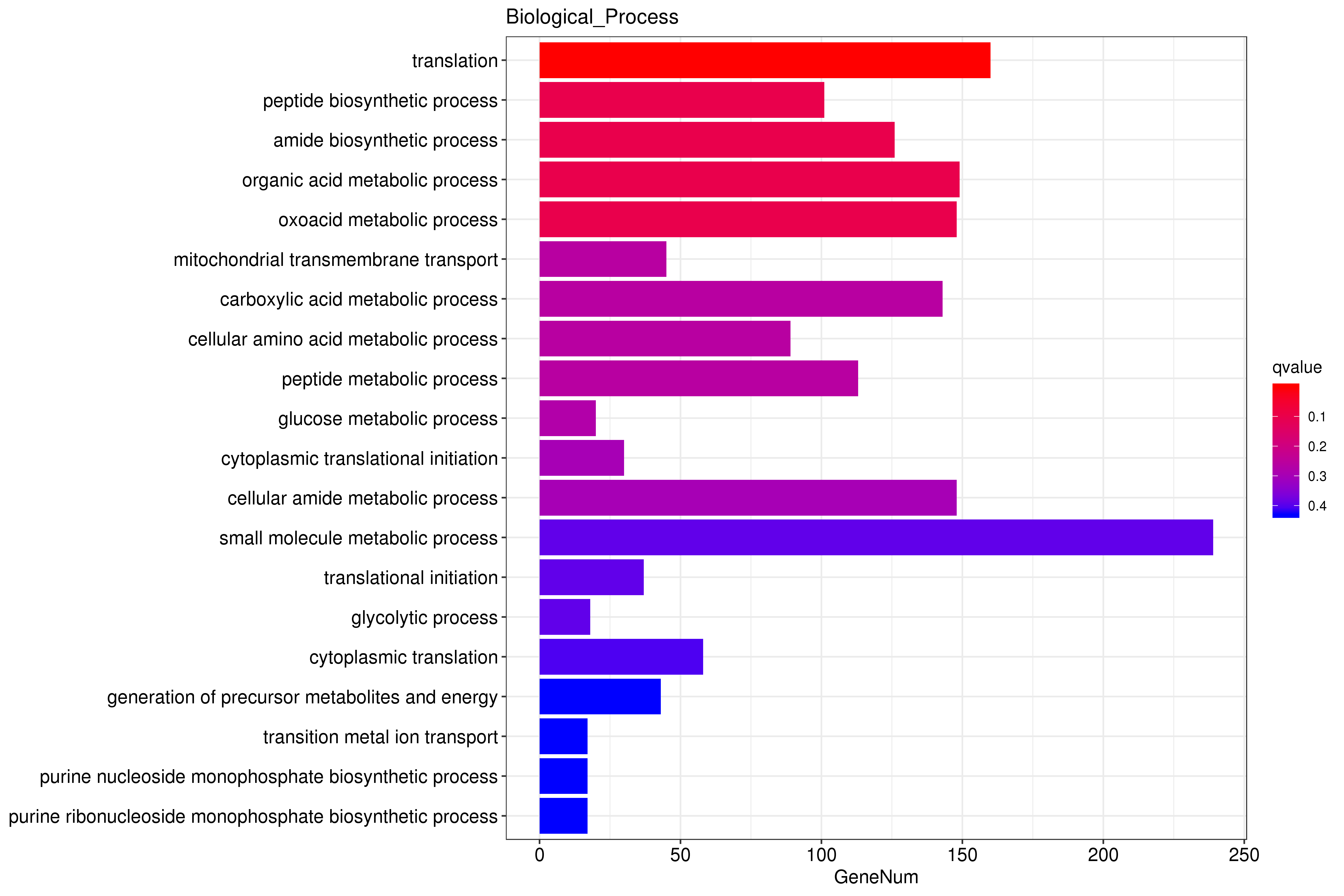


Fig. S1 KEGG (a) and GO (b) enrichment analysis showed the potential functions of peak-associated genes in the control group.

a


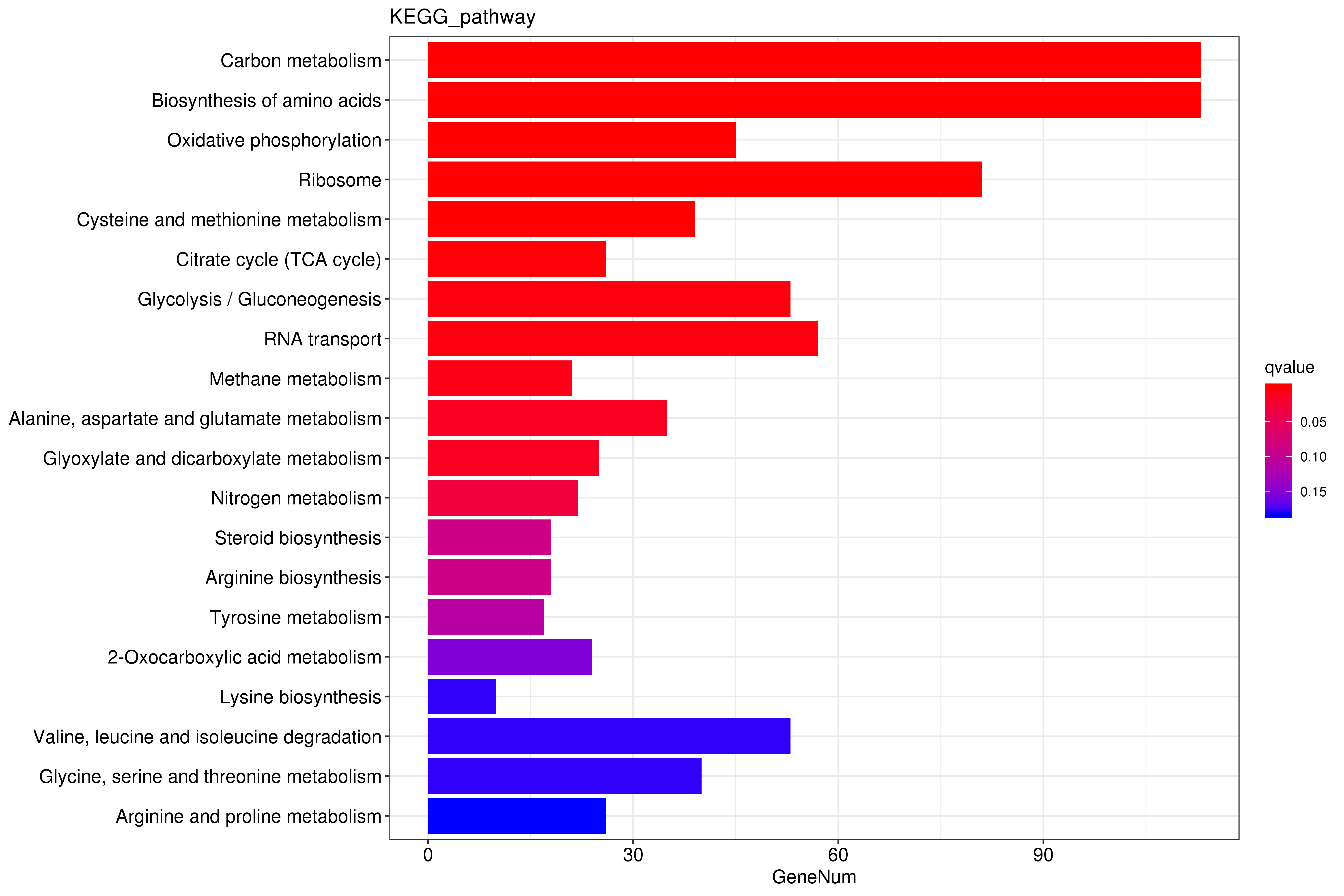


b


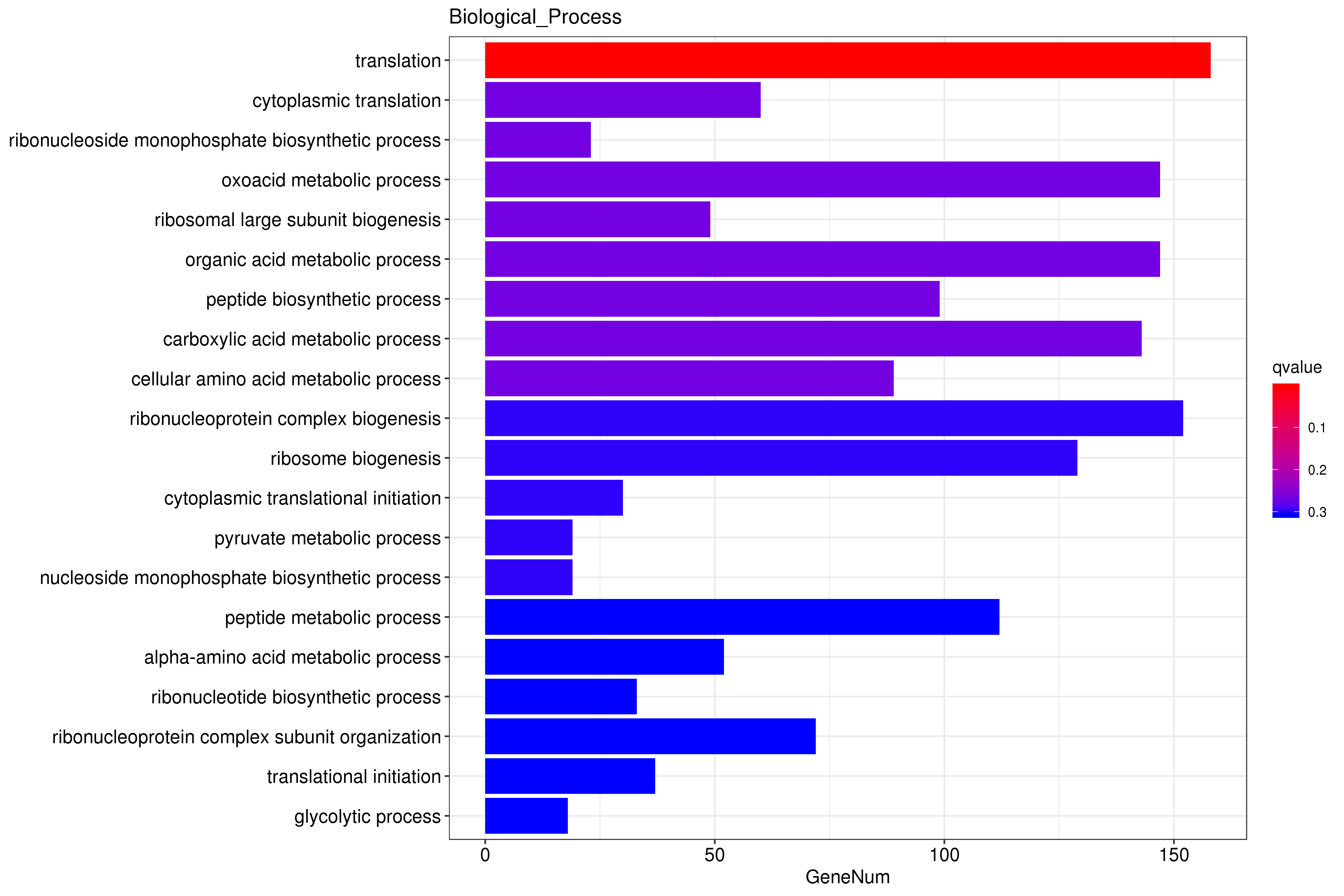


Fig. S2 KEGG (a) and GO (b) enrichment analysis showed the potential functions of peak-associated genes in the treatment group.

a


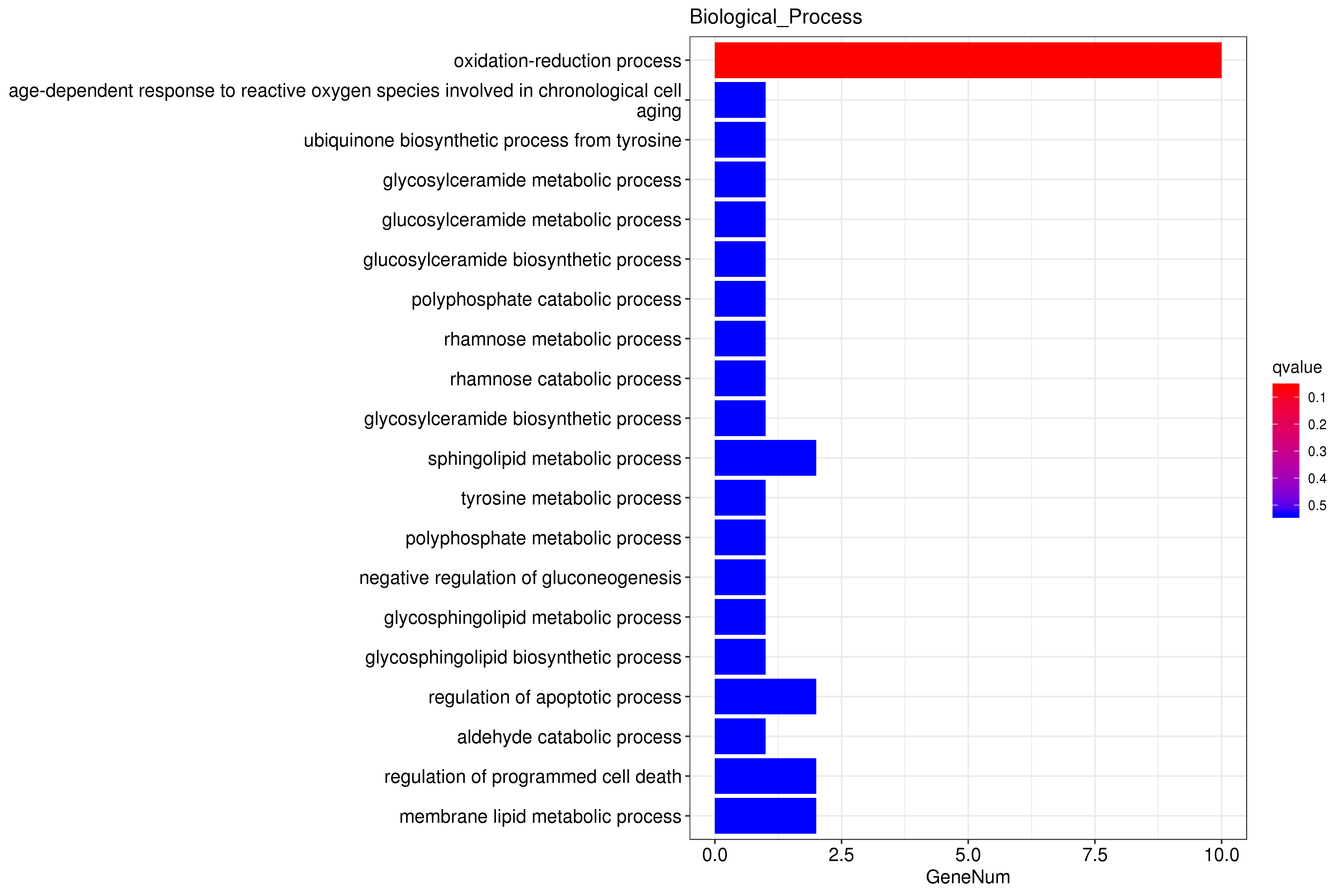


b


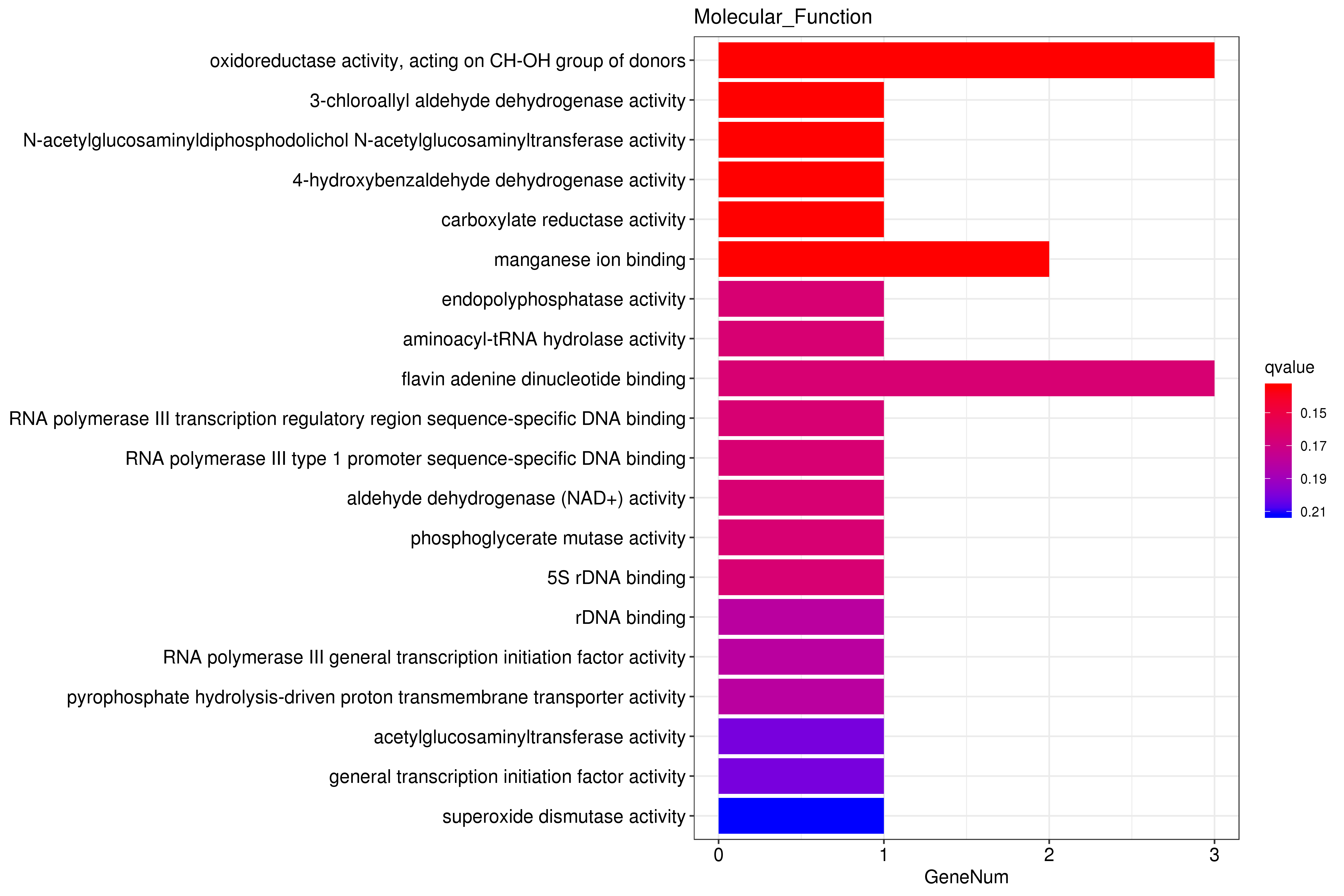


Fig. S3 GO enrichment in biological process (a) and molecular function (b) terms analysis showed the potential functions of DARs-associated genes between control and treatment group.


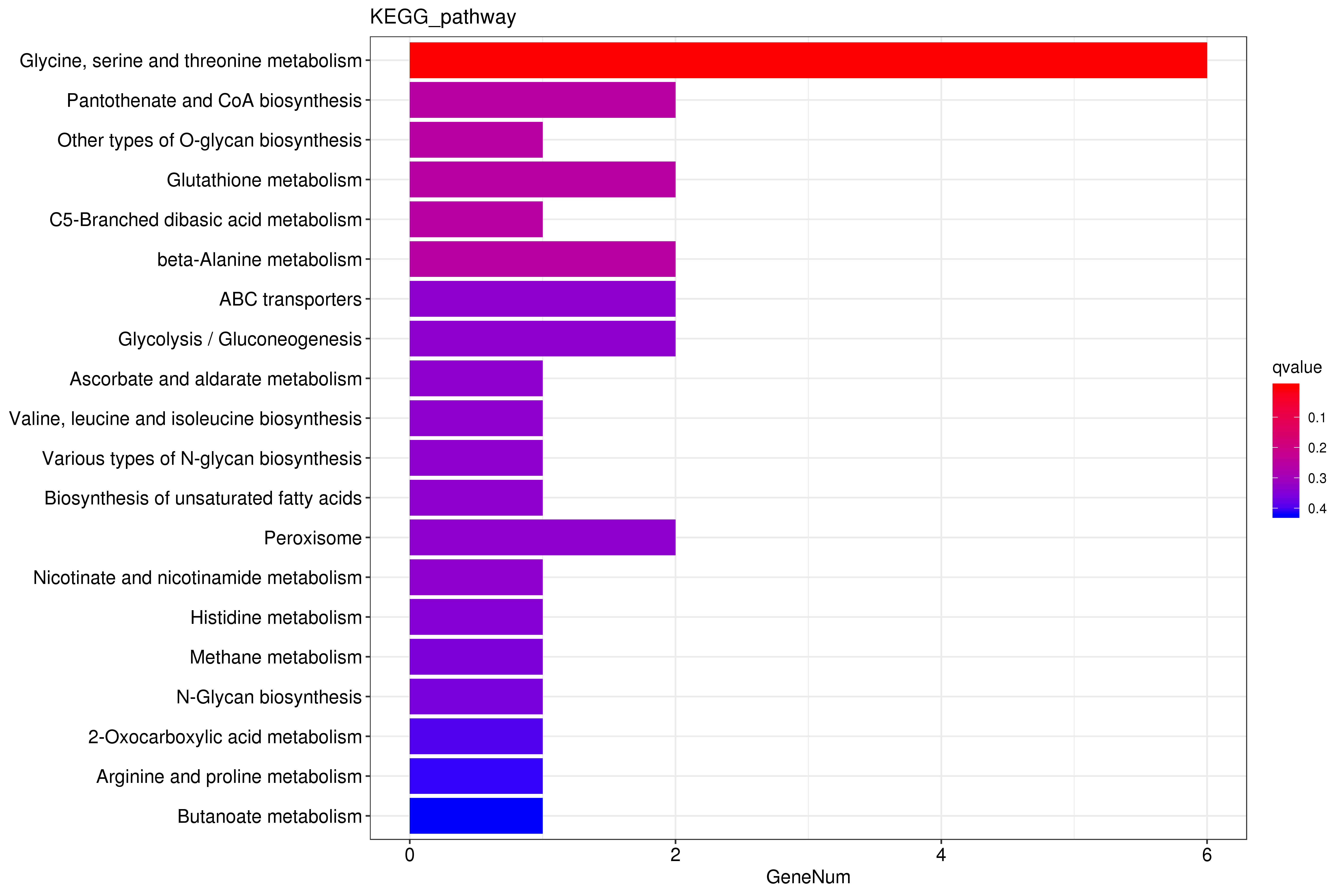


Fig. S4 KEGG enrichment analysis showed the potential functions of DARs-associated genes between control and treatment group.


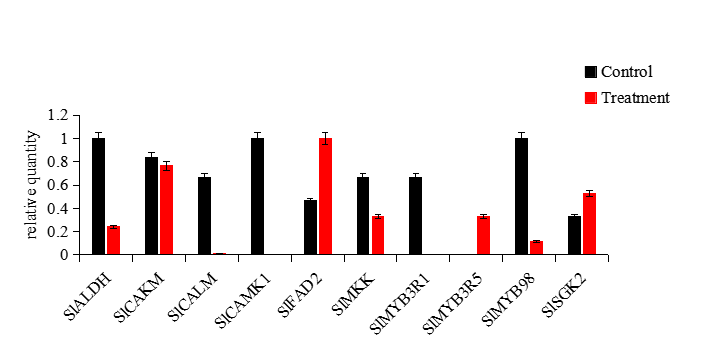


Fig. S5 Real-time quantitative RT-qPCR confirmation of *SlFAD2*, *SlALDH*, *SlMYB98*, *SlMKK*, *SlCALM*, *SlCAMK1*, *SlMYB3R1*, *SlMYB3R5*, *SlSGK2*, and *SlCALM*. Relative gene expressions were analyzed using the 2^−ΔΔCt^ method. Experiments were performed in triplicate. Error bars indicate standard deviation.

.
